# Somatic *RAP1B* gain-of-function variant underlies isolated thrombocytopenia and immunodeficiency

**DOI:** 10.1172/JCI169994

**Published:** 2024-07-11

**Authors:** Marta Benavides-Nieto, Frédéric Adam, Emmanuel Martin, Charlotte Boussard, Chantal Lagresle-Peyrou, Isabelle Callebaut, Alexandre Kauskot, Christelle Repérant, Miao Feng, Jean-Claude Bordet, Martin Castelle, Guillaume Morelle, Chantal Brouzes, Mohammed Zarhrate, Patricia Panikulam, Nathalie Lambert, Capucine Picard, Damien Bodet, Jérémie Rouger-Gaudichon, Patrick Revy, Jean-Pierre de Villartay, Despina Moshous

**Affiliations:** 1 Université Paris Cité, Paris, France.; 2 Imagine Institute, Laboratory of Genome Dynamics in the Immune System, Equipe Labellisée Ligue Contre le Cancer, Ligue 2023, INSERM UMR 1163, Paris, France.; 3 General Pediatrics–Infectious Diseases and Internal Medicine, Hôpital Robert Debré, Assistance Publique-Hôpitaux de Paris (AP-HP) Nord, Paris, France.; 4 INSERM UMR S 1176, Laboratory for Hemostasis, Inflammation and Thrombosis (HITh), Université Paris-Saclay, Le Kremlin-Bicêtre, France.; 5 Laboratory Lymphocyte Activation and Susceptibility to EBV infection, INSERM UMR 1163, Imagine Institute, Paris, France.; 6 Pediatric Immunology, Hematology and Rheumatology, Necker-Enfants Malades University Hospital, AP-HP, Paris, France.; 7 Laboratory Immunogenetics of Pediatric Autoimmune Diseases, INSERM UMR 1163, Imagine Institute, Paris, France.; 8 Biotherapy Clinical Investigation Center, AP-HP, Paris, France.; 9 Laboratory Human Lymphohematopoiesis, INSERM UMR 1163, Imagine Institute, Paris, France.; 10 Sorbonne University, Muséum National d’Histoire Naturelle, UMR CNRS 7590, Institut de Minéralogie, de Physique des Matériaux et de Cosmochimie, IMPMC, Paris, France.; 11 Laboratoire d’Hémostase, Centre de Biologie Est, Hospices Civils de Lyon, Bron, France.; 12 Laboratory of Onco-Hematology, Necker-Enfants Malades University Hospital, AP-HP, Paris, France, and INSERM U1151, Institut Necker-Enfants Malades, Paris, France.; 13 Genomics Core Facility, Institut Imagine-Structure Fédérative de Recherche Necker, INSERM U1163 and INSERM US24/CNRS UAR3633, Paris Descartes Sorbonne Paris Cité University, Paris, France.; 14 Laboratory “Molecular basis of altered immune homeostasis,” INSERM UMR 1163, Imagine Institute, Paris, France.; 15 Study Center for Primary Immunodeficiencies, Necker-Enfants Malades University Hospital, AP-HP, Paris, France.; 16 Centre de Référence des Déficits Immunitaires Héréditaires (CEREDIH), Necker-Enfants Malades University Hospital, AP-HP, Paris, France.; 17 CHU de Caen Normandie, Onco-Immunohématologie Pédiatrique, Caen, France.

**Keywords:** Hematology, Immunology, Cell migration/adhesion, Genetic diseases, Platelets

## Abstract

The ubiquitously expressed small GTPase Ras-related protein 1B (*RAP1B*) acts as a molecular switch that regulates cell signaling, cytoskeletal remodeling, and cell trafficking and activates integrins in platelets and lymphocytes. The residue G12 in the P-loop is required for the RAP1B-GTPase conformational switch. Heterozygous germline *RAP1B* variants have been described in patients with syndromic thrombocytopenia. However, the causality and pathophysiological impact remained unexplored. We report a boy with neonatal thrombocytopenia, combined immunodeficiency, neutropenia, and monocytopenia caused by a heterozygous de novo single nucleotide substitution, c.35G>A (p.G12E) in *RAP1B*. We demonstrate that G12E and the previously described G12V and G60R were gain-of-function variants that increased RAP1B activation, talin recruitment, and integrin activation, thereby modifying late responses such as platelet activation, T cell proliferation, and migration. We show that in our patient, G12E was a somatic variant whose allele frequency decreased over time in the peripheral immune compartment, but remained stable in bone marrow cells, suggesting a differential effect in distinct cell populations. Allogeneic hematopoietic stem cell transplantation fully restored the patient’s hemato-immunological phenotype. Our findings define monoallelic *RAP1B* gain-of-function variants as a cause for constitutive immunodeficiency and thrombocytopenia. The phenotypic spectrum ranged from isolated hematological manifestations in our patient with somatic mosaicism to complex syndromic features in patients with reported germline *RAP1B* variants.

## Abstract 2

Heterozygous RAP1B gain-of-<wbr></wbr>function variants alter integrin-mediated platelet activation and lymphocyte trafficking, with mosaicism resulting in isolated thrombocytopenia and immunodeficiency while germline mutations involve multiple tissues.

## Graphical abstract

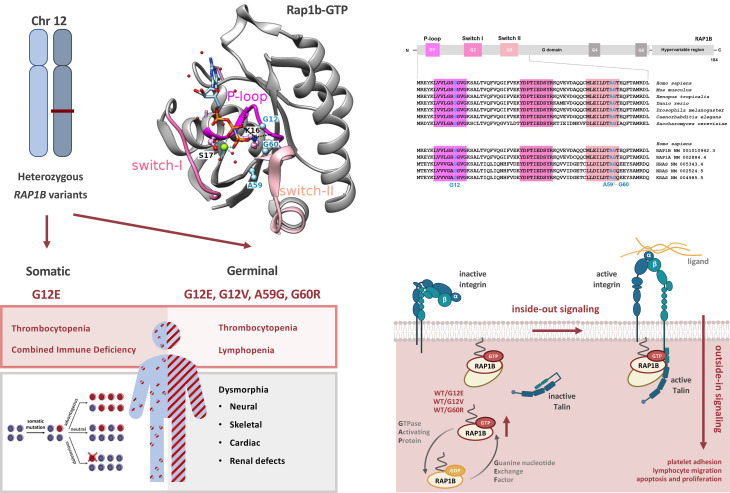

## Introduction

Thanks to next-generation sequencing (NGS) approaches, the list of genetic syndromes that cause thrombocytopenia has grown in recent years and now includes over 40 genes (1). However, there are still patients with presumed constitutional thrombocytopenia who lack a molecular etiology despite extensive genetic testing. Within this group, an increasing number of human pathologies have highlighted the interplay between the actin cytoskeleton and Ras homology (Rho) GTPases, signaling proteins with crucial roles in triggering multiple immune functions. Through their interactions with a broad range of effectors and kinases, they regulate cytoskeletal dynamics, cell polarity, and immune cell trafficking and proliferation. Defects in Rho GTPases lead to various immune pathologies, including combined immune deficiencies (2). Variants in genes encoding Rho GTPase effectors, such as MYH9, a member of the ARP2/3 complex, WASP, and DIAPH1, are associated with inherited human thrombocyte disorders (3), Wiskott-Aldrich syndrome and its clinical triad comprising microthrombocytopenia, eczema, and immunodeficiency being the most prominent example (4).

Here we report a patient with neonatal thrombocytopenia and bleeding diathesis associated with neutropenia, monocytopenia, and combined immunodeficiency (CID). Whole-exome sequencing (WES) analysis identified a monoallelic de novo RAS-related protein 1b (*RAP1B*) variant, a gene encoding a factor of the cytoskeleton/Rho-GTPase network. Heterozygous *RAP1B* germline variants have been recently described in patients with syndromic thrombocytopenia (ST) associated with multiple clinical features ranging from facial dysmorphia, microcephaly, and neurodevelopmental delay to organ involvement, particularly brain, heart, kidney, and skeletal abnormalities (5, 6). While these patients developed mild neutro- and lymphopenia, they had no clinical history suggesting an associated immunodeficiency. However, neither their immune nor platelet function had been investigated. It has been speculated that germline gain-of-function (GOF) *RAP1B* variants may be causative, but so far, no comprehensive functional studies have been performed.

Our study provides evidence that the *RAP1B* variant in our patient as well as two recently described *RAP1B* variants in patients with ST were GOF variants impairing platelet activation. Thus, our study defines monoallelic GOF *RAP1B* variants as cause for primary immunodeficiency associated with thrombocytopenia.

## Results

### Clinical and immune-hematological phenotype.

**Figure 1:**
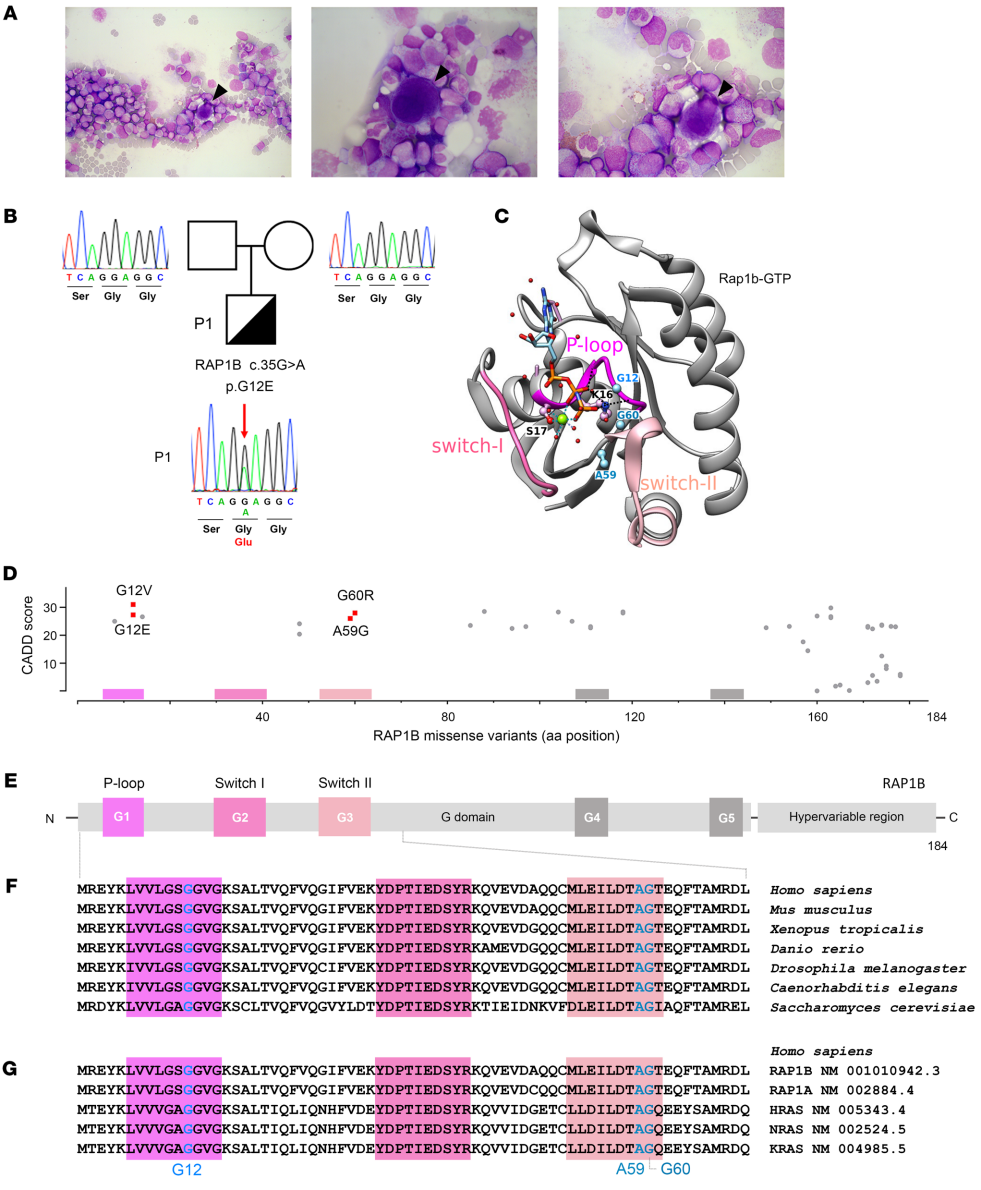
*RAP1B* variants and RAP1B protein structure. (**A**) May-Grünwald-Giemsa staining of P1 BM smear at age of M18 showing reduced richness for the patient’s age, elements at all stages of maturation, predominance of the granular lineage, absence of atypical cells, and presence of rare hypolobed megakaryocytes (black arrowheads). Original magnification, ×500 (left); ×1,000 (center and right). (**B**) P1 pedigree and familial segregation. Sanger sequencing of *RAP1B* in whole peripheral blood from P1 and his parents shows the heterozygous *RAP1B* c.35G>A (p.G12E) variant in P1 (red arrow), but not in P1’s parents, confirming its de novo nature. (**C**) Ribbon representation of the 3D structure of rat Rap1B bound to a nonhydrolyzable GTP analog (GppNHp, pdb 3X1X) (42). The sequences of rat Rap1B and human RAP1B are identical, except for C139 in the human sequence, which is replaced by serine in the rat sequence. This surface residue is far from the nucleotide-binding site. P-loop, switch I, and switch II regions are shown in pink. Magnesium ion is shown in green, water molecules in red, and the residues G12, A59, and G60, which have been found mutated in patients (Table 3), are in blue. (**D**) CADD score and amino acid position of all human RAP1B missense variants listed in gnomAD (11) as of February 6, 2023. RAP1B variants reported in patients are shown in red: G12E (P1) and G12V, A59G, and G60R (P2, P3, and P4) (5, 6). (**E**) Schematic representation of the secondary structure of human RAP1B with G domain–containing P-loop (G1), switch I and switch II (G2 and G3), G4, and G5 functional domains (33, 61), and hypervariable region. (**F**) Multiple sequence alignment of RAP1B G1–G3 functional domains from different species (62). (**G**) Multiple sequence alignment of G1–G3 functional domains of human small GTPases: RAP1B, RAP1A, HRAS, NRAS, and KRAS (62).

A full-term eutrophic boy (patient 1 [P1]), born to nonrelated White parents, presented neonatal petechiae without any other clinical features. Whole blood count (WBC) revealed severe thrombocytopenia, lymphopenia, neutropenia, and monocytopenia (Table 1). At 1 week of age, bone marrow (BM) smear was hypocellular, displaying rare megakaryocytes. BM biopsy at the age of 3 months (M3) showed megakaryocyte maturation defects and granular hypoplasia, while BM immune phenotype and karyotype were normal. Extensive etiological investigations were negative (Supplemental Table 1; supplemental material available online with this article; https://doi.org/10.1172/JCI169994DS1). Intriguingly, hemorrhagic manifestations were more important than expected, given platelet counts requiring repeated platelet transfusions. At M8, neutropenia persisted. Hypogammaglobulinemia and low CD4^+^, CD8^+^, and naive T cell counts (Table 2) were consistent with CID. However, P1 did not suffer from severe infections under antimicrobial prophylaxis (trimethoprim-sulfamethoxazole, valacyclovir) and immunoglobulin substitution. BM smear at M18 showed decreased cellularity for age and only rare, abnormal, hypolobed megakaryocytes (Figure 1A).

Given the severe immunohematological condition, P1 received a 10/10 HLA allele-matched unrelated hematopoietic stem cell transplantation (HSCT) at the age of 2 years after myeloablative conditioning with busulfan, fludarabine, and antithymocyte globulin. He achieved 100% donor chimerism and excellent hematological and immunological reconstitution (Table 1 and Table 2). At last follow-up 62 months after HSCT, P1, aged 7.2 years, was alive and well and off treatment.

Overall, the association of severe early onset thrombocytopenia, neutropenia, monocytopenia, and CID, corrected by allogenic HSCT, was consistent with an inborn hematopoietic and immune disorder.

### P1 carries a monoallelic de novo RAP1B c.35G>A p.G12E variant.

WES on genomic DNA (gDNA) from PBMCs revealed the heterozygous single nucleotide substitution c.35G>A in the ubiquitously expressed *RAP1B* gene (NCBI ID, NM_001010942.3, Chr12:68648759) causing p.G12E in P1, but not in his parents. Sanger sequencing confirmed that this variant was de novo (Figure 1B). *RAP1B* RNA was present in P1 PBMCs, which carried the c.35G>A variant in a heterozygous state. We considered RAP1B a strong candidate, as its GTP-bound active form mediates integrin activation in platelets and lymphocytes (7–10). RAP1B-G12E variant (hereafter referred to as G12E) was absent in public (Genome Aggregation Database [gnomAD]) (11) and in-house databases and had not been described in patients, to our knowledge, at the time we started our investigations. It was predicted deleterious according to in silico tools including Combined Annotation Dependent Depletion (CADD) score (12) (score 31), PolyPhen-2 (13) (score 1), and Sorting Intolerant From Tolerant (SIFT) (14) (score 0). G12 is located in the RAP1B active site within the P-loop (Figure 1C), in which only 2 other missense variants have been reported in gnomAD (11) (Figure 1, D and E). RAP1B is highly evolutionary conserved from yeast to humans (Figure 1F) and among other human GTPases (Figure 1G).

### RAP1B c.35G>A is a somatic variant in P1.

WES raw data revealed that among the 257 reads covering the region of interest, only 108 (42%) carried the *RAP1B* c.35G>A variant in P1 gDNA from PBMCs at M12. This suggested a slight imbalance between WT and mutated alleles. Sanger sequencing showed that P1 primary fibroblasts did not carry the variant, while microsatellite analysis confirmed their identity (Supplemental Figure 1, A and B), arguing for a somatic mutation.

**Figure 2:**
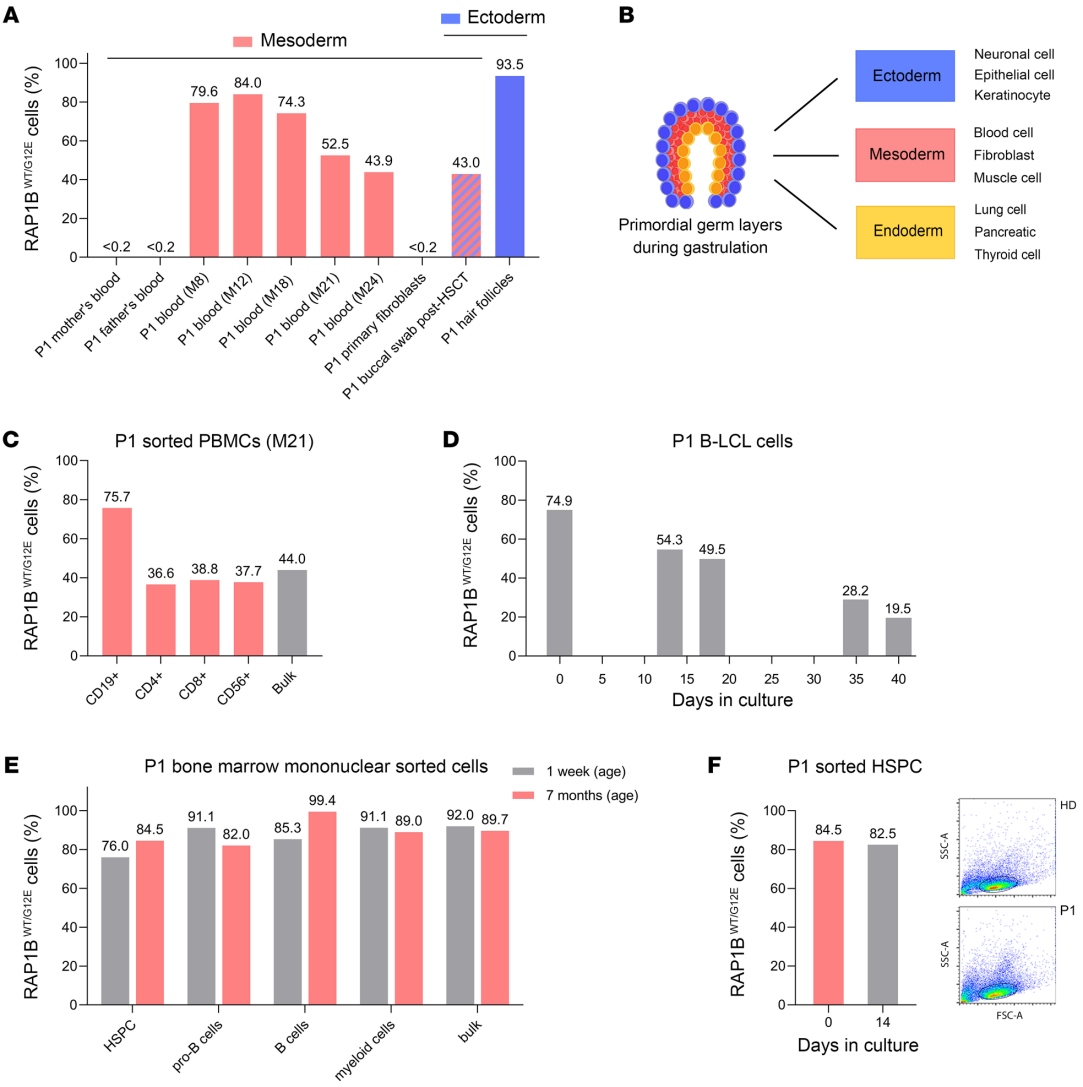
Characterization of P1 RAP1B G12E somatic variant. (**A**) Proportion of RAP1B^WT/G12E^ cells (%) in gDNA samples determined by *RAP1B* c.35G > A (RAP1B G12E) VAF analysis using NGS (Supplemental Table 2). Absence of RAP1B^WT/G12E^ cells in P1’s parents’ peripheral blood (16). RAP1B^WT/G12E^ cells are absent in P1 mesoderm-derived primary fibroblasts, but present in P1 mesoderm–derived peripheral blood (before HSCT), ectoderm-derived hair follicles, and buccal swab (obtained 7 months after HSCT). (**B**) Schematic representation of the process of gastrulation generating the 3 primary germ layers (ectoderm, endoderm, mesoderm). (**C**) Quantification of RAP1B^WT/G12E^ cells (%) using NGS in P1 PBMCs (at M21) sorted into CD19^+^ B cells, CD3^+^CD4^+^ T cells, CD3^+^CD8^+^ T cells, and CD56^+^ NK cells (Supplemental Table 2). (**D**) Quantification of RAP1B^WT/G12E^ cells (%) in P1 B-LCL cells cultured over 40 days. B-LCL cells were established from P1 PBMCs obtained at M18. VAF was analyzed using EditR (63) (Supplemental Table 2). (**E**) Quantification of RAP1B^WT/G12E^ cells (%) using NGS in sorted P1 BM mononuclear cells harvested at the ages of 1 week and M7: B cells (CD45^+^CD34^–^CD19^+^), pro–B cells (CD45^+^CD34^+^ CD19^+^), myeloid cells (CD45^+^CD11b^+^), and hematopoietic stem progenitor cells (HSPCs) (CD45^+^CD34^+^CD19^–^) (Supplemental Table 2). (**F**) Quantification of RAP1B^WT/G12E^ cells (%) by NGS (Supplemental Table 2) in sorted P1 HSPCs harvested at M7 after 14 days in culture. FACS images of P1 and control HSCP cells after 14 days in culture. numbers above bars indicate the percentages of RAP1B^WT/G12E^ cells.

Variant allele frequency (VAF) analysis by high-throughput sequencing in different cell types from P1 (Supplemental Table 2) indicated that all PBMC samples prior to HSCT as well as hair follicle and buccal swab cells (obtained 7 months after HSCT) carried the *RAP1B* c.35G>A variant (Figure 2A). As buccal swab may not contain only recipient oral mucosal epithelial cells, but also donor-derived circulating leukocytes from saliva, VAF in buccal swab may be underestimated. NGS confirmed that the *RAP1B* variant was absent in P1 primary fibroblasts and both parents’ peripheral blood (Figure 2A), with VAFs below the established cut-off for somatic variants (15, 16).

Collectively, these results indicate that *RAP1B* c.35G>A is a de novo somatic variant, present in cells and tissues originating from different embryonic layers (Figure 2, A and B) such as ectoderm (oral mucosal epithelial cells and hair follicles) and mesoderm (hematopoietic cells), but absent in mesoderm-derived primary fibroblasts.

### VAF in P1 PBMCs and cultured cells decreased over time, but remained stable in BM cells.

The earliest available DNA sample from peripheral blood at M8 revealed a VAF of 39.8% corresponding to 79.6% cells carrying the *RAP1B* c.35G>A variant in a heterozygous state, compared with 43.9% at M24 (Figure 2A), suggesting a deleterious effect of the somatic variant, with progressive loss of cells carrying this variant in peripheral blood. There were no significant WBC modifications (Table 1) concomitant to this decrease. We studied the percentages of *RAP1B* c.35G>A positive cells in different FACS-sorted immune subpopulations. At M21, 75.7% of CD19^+^ B cells carried the variant, but only 36.6% to 38.8% of CD4^+^, CD8^+^, and NK cells (Figure 2C), suggesting that *RAP1B* c.35G>A had a distinct deleterious impact on different cell populations. The T cell receptor (TCR) rearrangement profiles of P1 T lymphocytes at M8, M12, and M23 did not show major differences, arguing against a clonal selection process underlying the decreased VAF in the T cell compartment (data not shown).

We next studied the VAF in P1 B lymphoblastoid cell line (B-LCL) cells. The percentages of B-LCL cells carrying the variant progressively decreased from 74.9% to 19.5% after 40 days of culture (Figure 2D), suggesting a counterselection of B-LCL cells carrying *RAP1B* c.35G>A, as observed in peripheral blood (Figure 2A). In contrast to the progressive decrease of the variant in P1 peripheral blood cells (Figure 2A), the percentages remained stable in BM samples obtained at age 1 week and M7 (Figure 2E), arguing against a deleterious impact of the variant in this cell compartment. To confirm this observation, we sorted and cultured P1 CD34^+^ cells for 2 weeks as previously described (17). We did not observe any significant decrease in *RAP1B* c.35G>A VAF (Figure 2F).

### RAP1B-G12E is associated with severe thrombocytopenia and abnormal platelet function.

**Figure 3:**
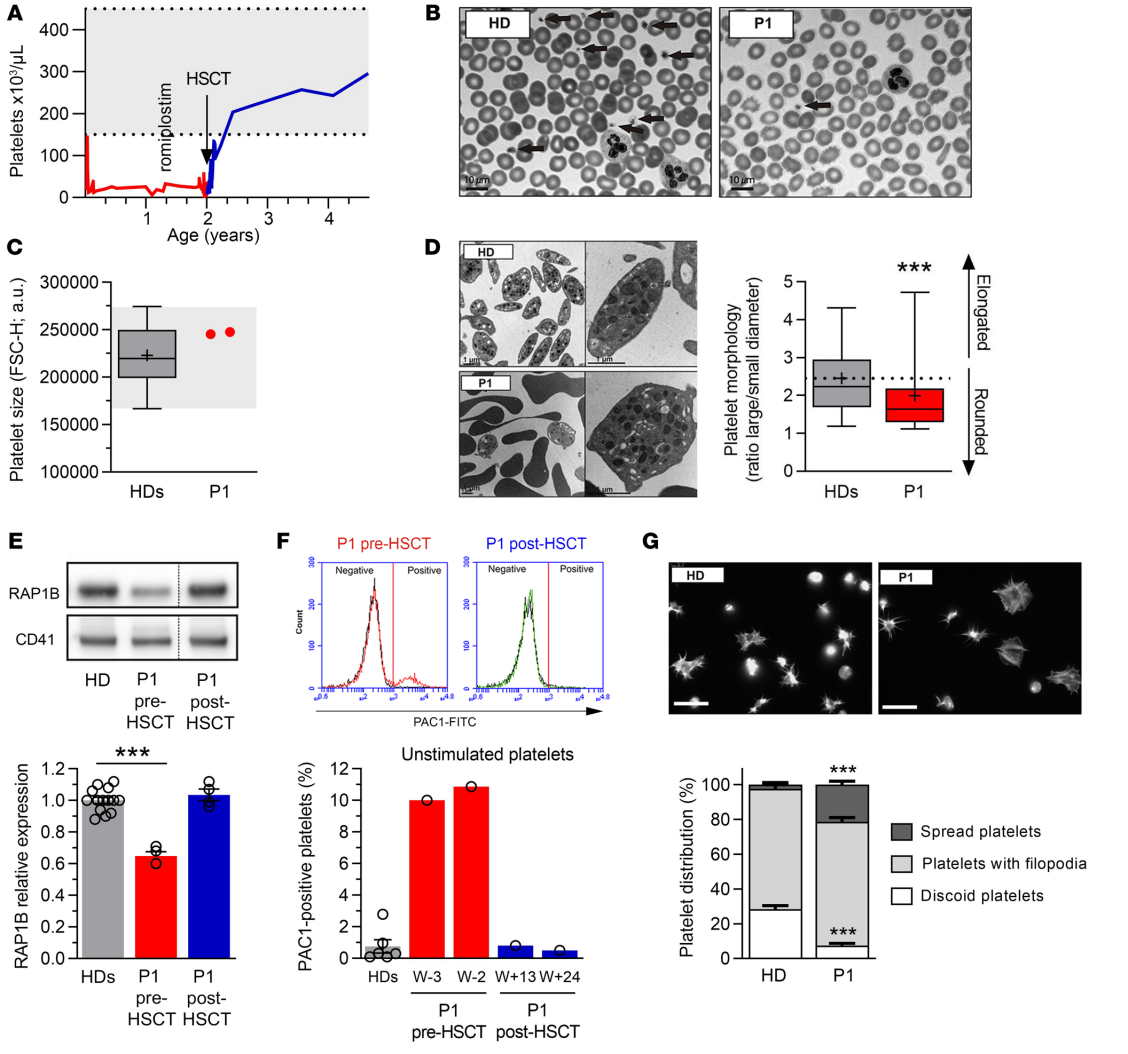
Characterization of P1 platelets. (**A**) P1 platelet counts before and after HSCT. (**B**) Representative blood smears of HD and P1 after May-Grünwald-Giemsa staining. Arrows indicate platelets. (**C**) Platelet size evaluated by flow cytometry. Each dot represents the mean forward scatter (FSC-H) of washed platelets. HDs, *n* = 65; P1, *n* = 2. (**D**) Platelet ultrastructure analyzed by TEM. The box-and-whisker plots represent platelet morphology (*n* = 100) defined by the ratio between the large and the small cell diameter. In **C** and **D**, whiskers represent the 5th to 95th percentiles, the box corresponds to the interquartile range, the center line indicates the median, and the cross indicates the mean. (**E**) RAP1B expression evaluated by Western blotting (representative of at least 4 experiments). Graph (lower panel) represents RAP1B expression after normalization by CD41 expression. Data are represented as mean of the relative expression (HDs set to 1) ± SEM. HDs, *n* = 14; P1, before HSCT and after HSCT, *n* = 4. Dashed lines indicate that the samples were derived from the same gel but were noncontiguous. (**F**) Integrin α_IIb_β_3_ activation evaluated in unstimulated platelets by flow cytometry using PAC1 antibody, which recognizes the active conformation of the integrin. The cytograms represent the traces of HD (black line) and of P1 before (red) and after HSCT (green). Graph (lower panel) represents the percentage ± SEM of PAC1-positive platelets. HDs, *n* = 6; P1, *n* = 1. W, weeks before HSCT; W+, weeks after HSCT. (**G**) Spreading of HD and P1 platelets before HSCT onto fibrinogen matrix analyzed by epifluorescence microscopy using fluorescently labeled phalloidin. Graph (lower panel) represents the percentage of discoid platelets (white), platelets with filopodia (light gray), and spread platelets (dark gray). Scale bars: 10 μm. Statistical significance was determined for **D** by 2-tailed Student’s *t* test and for **E** and **G** by 1-way ANOVA with Dunnett’s multiple-comparisons test. ***P < 0.001.

Since RAP1B is essential to platelet function (8, 18, 19), we next studied P1 platelets. From birth until HSCT, P1 presented platelet counts ranging between 15,000 and 30,000/μL, also when treated with the thrombopoietin receptor agonist romiplostim (Figure 3, A and B). P1 platelets had normal size, as evaluated by flow cytometry (Figure 3C) and automated platelet counter (Table 1). However, ultrastructure analysis by transmission electron microscopy (TEM) revealed abnormal platelet morphology, with platelet shape being more round than discoid (Figure 3D). RAP1B protein expression in P1 platelets was decreased by 35% compared with controls (*P* < 0.001), but recovered normal levels following HSCT (Figure 3E).

Due to P1’s young age and severe thrombocytopenia, extensive studies of platelet function were not feasible. The activation of integrin α_IIb_β_3_ is essential for platelet aggregation. We evaluated its activation by flow cytometry using PAC1 antibody, which recognizes its active conformation (Figure 3F). P1 had a distinct platelet population (10%) with unusual integrin α_IIb_β_3_ activation in unstimulated conditions, confirmed in 2 independent experiments. After HSCT, α_IIb_β_3_ activation in P1 resting platelets was normal. To confirm this unusual integrin activation, we plated unstimulated washed platelets onto fibrinogen matrix in order to analyze platelet morphology (Figure 3G). In this assay, the engagement of activated integrin α_IIb_β_3_ with fibrinogen, its ligand, induces an outside-in signaling responsible for platelet activation, cytoskeleton reorganization, and morphological changes, including membrane extensions named filopodia occurring prior to full platelet spreading. In the control, 28.4% ± 2.0% platelets showed a discoid shape (corresponding to resting platelets) and only 2.4% ± 1.2% platelets a spread shape (corresponding to platelets at the last step of activation). While P1 had a significant decrease in the percentage of nonactivated platelets (7.5% ± 1.1% discoid platelets, *P* < 0.001), for the benefit of fully activated ones (21.4% ± 2.0% spread platelets; *P* < 0.001), there was no overexpression of integrin α_IIb_β_3_ or other key platelet receptors such as glycoprotein (GP)VI, GPIbα, or GPIX (data not shown). Together, these results confirm the presence of an unusual spontaneously active conformation of integrin α_IIb_β_3_ in P1 unstimulated platelets.

### RAP1B-G12E is a GOF variant.

To explore the impact of RAP1B-G12E in P1 cells, we performed migration assays. P1 primary fibroblasts migrated normally (Supplemental Figure 1C), a result consistent with the absence of the *RAP1B* variant in these cells. We next evaluated spontaneous and SDF-1α–mediated in vitro migration of freshly isolated PBMCs. SDF-1α (or CXCL12) chemoattractant is the canonical ligand of the CXCR4 receptor. The activation of the CXCL12/CXCR4 axis is involved in migration, chemotaxis, and proliferation in lymphocytes (20) and contributes, through inside-out signaling, to increase lymphocyte integrin affinity (21). Fibronectin is the ligand of VLA-4 (α_4_/β_1_ CD49d/CD29) integrin. Its interaction with the high-affinity integrin could, in turn, through outside-in signaling, activate downstream cascades modifying actin dynamics and mediating cell migration (22).

**Figure 4:**
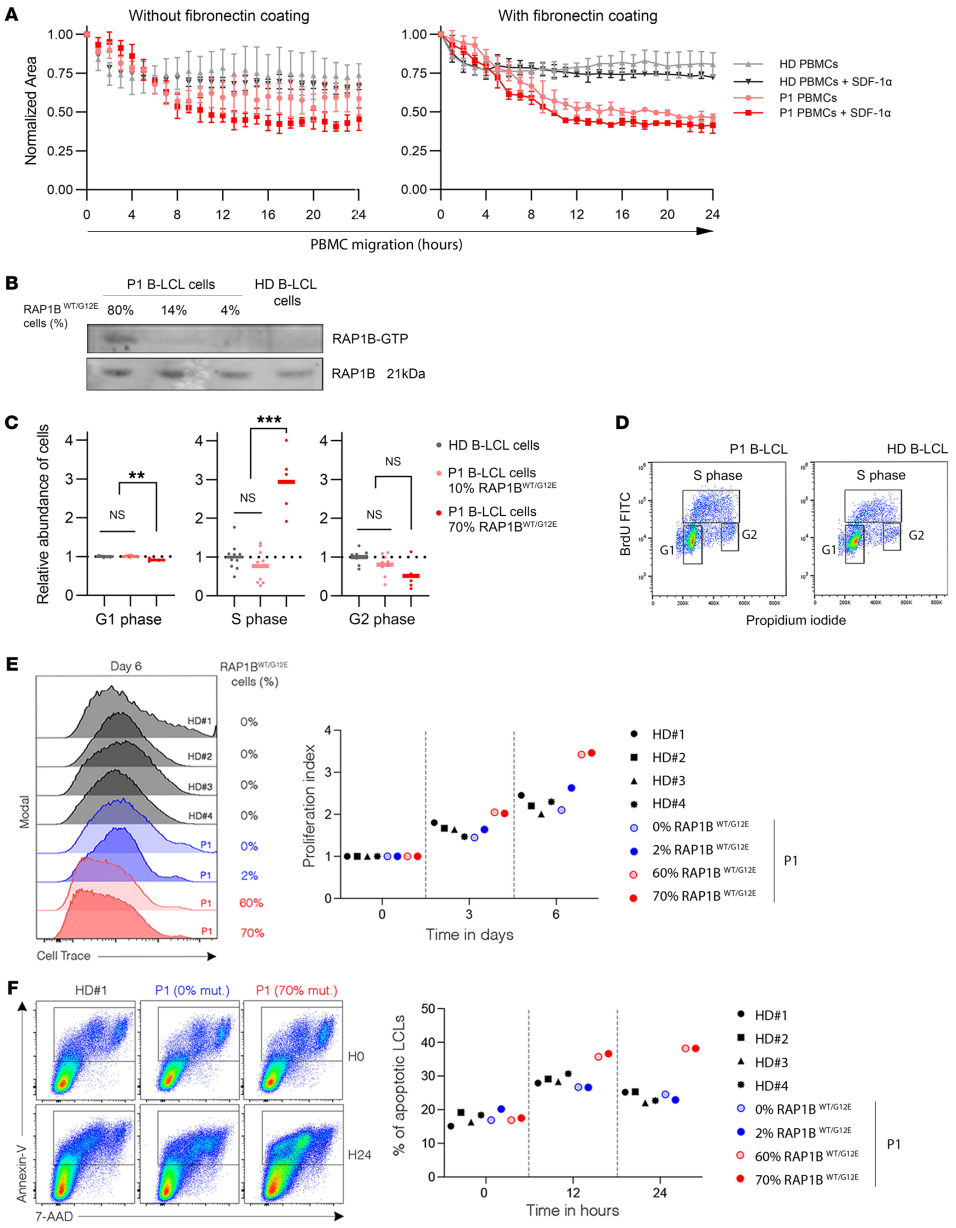
P1 lymphocytes functional analysis. (**A**) Analysis of P1 PBMC spontaneous and chemoattractant-induced migration in the absence (left) or presence (right) of fibronectin coating (100 mg/ml) using Transwell devices. Experiments were repeated twice in triplicates. One representative of 2 independent experiments is shown. SDF-1α (CXCL12, 1,500 ng/mL) was used as chemoattractant. Means of triplicates (with SD) are represented for patient and control cells in the graphs. At the time of the test, P1 PBMCs carried 52.5%RAP1B^WT/G12E^ cells. (**B**) RAP1B activation, calculated as the ratio RAP1B-GTP/RAP1B total expression, was evaluated in P1 and control B-LCL cells by Western blotting after pull-down assay. P1 B-LCL cell bulk populations used for the experiment contained 80%, 14%, and 4% RAP1B^WT/G12E^ cells. The blot is representative of 2 independent experiments. (**C**) Analysis of the relative proportion of P1 and control B-LCL cells in G1, S, and G2 cell-cycle phases after double staining with anti-BrdU monoclonal antibody and PI. P1 B-LCL cell bulk populations contained 70% and 10% RAP1B^WT/G12E^ cells. Two different healthy B-LCL cell unrelated controls (1 age matched) were used for normalization. Experiments were performed in triplicate for 2 independent experiments. The representative graph shows the gated populations. Statistical significance was determined by Mann-Whitney *U* test. **P < 0.01; ***P < 0.001. (**D**) Percentages of RAP1B^WT/G12E^ cells in sorted G1, S, and G2 phase populations after cell-cycle analysis. P1 B-LCL cell bulk population contained 70% RAP1B^WT/G12E^ cells. (**E**) Representative histograms(left) showing cell divisions by CellTrace Violet staining of synchronized HDs (gray histograms) and P1 (blue and red histograms) B-LCL cells after 6 days of culture. P1 B-LCL cell bulk contained 0% (light blue), 2% (dark blue), 60% (light red), or 70% (dark red) RAP1B^WT/G12E^ cells. Dot plots graph (right) showing index of proliferation of HD and P1 B-LCL cells calculated from FACS histograms shown in the left panel (with same color code) at indicated time of culture. Each symbol corresponds to 1 individual HD (black) or P1 (blue or red). Data are from 1 of 2 independent experiments. (**F**) Representative FACS dot plots (left) depicting annexin V and 7-AAD expressions of HD and P1 B-LCL cells containing 0% or 70% RAP1B^WT/G12E^ cells for 0 and 24 hours. Dot plot graph (right) showing apoptotic cells (annexin V^+^ and 7-AAD^+^) of HD and P1 B-LCL cells calculated from FACS dot plots shown in the left panel with color code as in **E** at indicated time of culture. Each symbol corresponds to 1 individual HD (black) or P1 (blue and red). Data are from 1 of 2 independent experiments.

P1 and control PBMCs showed no significant difference in cell migration assays. Nonetheless, upon fibronectin coating, P1 PBMCs migrated more than control cells in all conditions (Figure 4A), while WBC distribution, LFA-1, and VLA-4 integrin subunit expression were roughly similar in P1 and control PBMCs (Table 1 and data not shown). These findings suggest a basal overactivation of the VLA-4/fibronectin axis in P1 lymphocytes that may increase migration through outside-in signaling, supporting that RAP1B-G12E is a GOF variant.

To confirm this hypothesis, we analyzed RAP1B activity through pull-down assays in order to determine the amount of active GTP-RAP1B among total RAP1B protein. RAP1B activation was increased in P1 B-LCL cells containing 80% of RAP1B^WT/G12E^ cells compared with control and P1 B-LCL cell populations harboring low percentages of RAP1B^WT/G12E^ cells (Figure 4B), confirming that RAP1B-G12E is a GOF variant. P1 B-LCL RAP1B^WT/G12E^ showed increased percentages of cells in S-phase compared with healthy donor (HD) B-LCL cells (Figure 4, C and D). P1 B-LCL cells with high percentages of RAP1B^WT/G12E^ cells proliferated more than 4 HD B-LCL cells or P1 B-LCL cells with low percentages of RAP1B^WT/G12E^ (Figure 4E). P1 RAP1B^WT/G12E^ B-LCL cells in culture exhibited higher proportions of apoptotic cells compared with HD B-LCL cells (Figure 4F). Taken together, data for cell cycle, cell division, and cell apoptosis indicated enhanced proliferation in P1 RAP1B^WT/G12E^ B-LCL cells.

### B-LCL cells carrying the RAP1B-G12E variant present abnormal morphological features.

**Figure 5:**
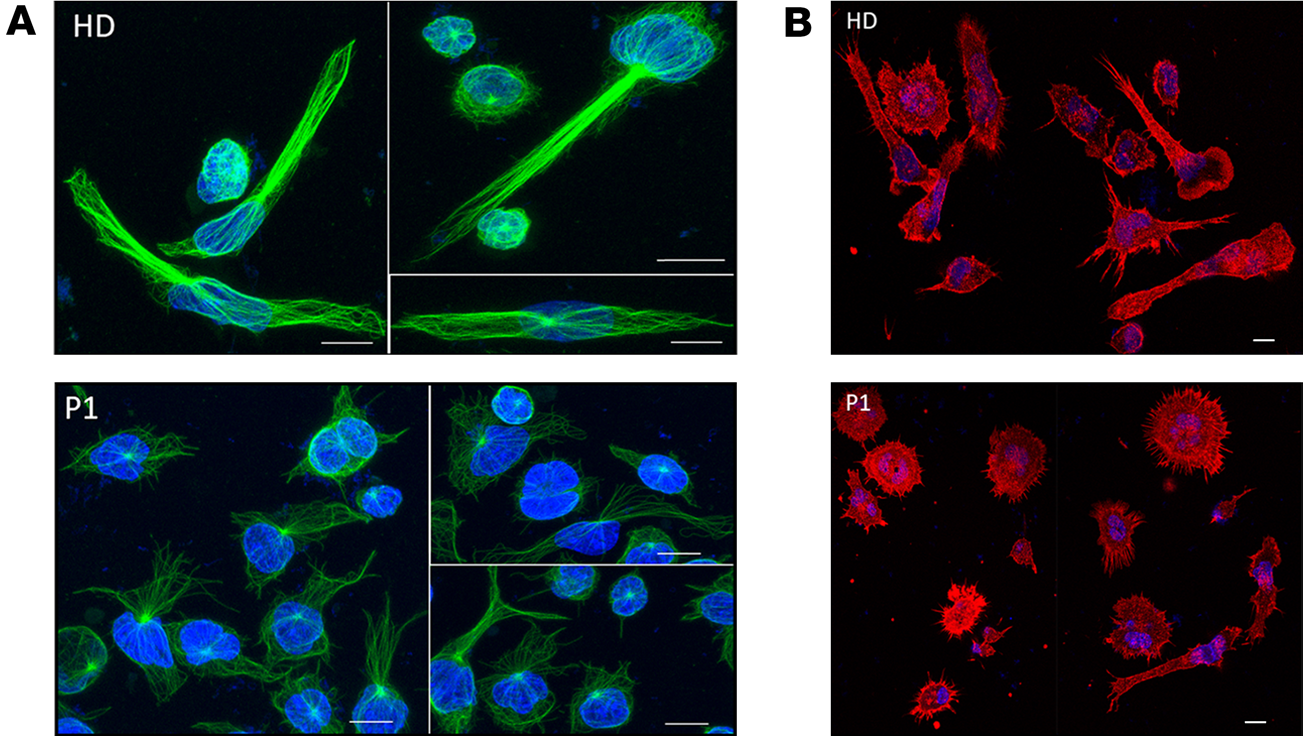
RAP1B G12E leads to altered cell morphology in B-LCL. (**A**) Altered microtubule organization of immortalized P1 B lymphoblastic cell lines after spreading. Green corresponds to microtubules staining. Blue corresponds to nucleus staining. Coverslips were coated with anti-CD44 antibody. Cells from P1 were rounder and had altered microtubule organization compared with HD cells. (**B**) Altered actin cytoskeleton in immortalized P1 B lymphoblastic cell lines after spreading. Red corresponds to actin staining. Blue corresponds to nucleus staining. P1 cells were more rounded than HD cells. Scale bars: 15 μm.

Given the observation that P1 PBMCs seem to migrate quite efficiently even without SDF-1α, we wanted to explore P1 cell morphology. It had been shown that activated Rap1 induced T cell polarity, also in the absence of chemokines (7, 23). When compared with immortalized B-LCL cells from HDs after spreading, P1 B-LCL cells showed altered microtubule organization (Figure 5A) as well as altered actin cytoskeleton (Figure 5B). P1 cells were also more rounded than HD cells.

### Functional studies in human cell lines confirm the GOF nature of RAP1B variants.

**Figure 6:**
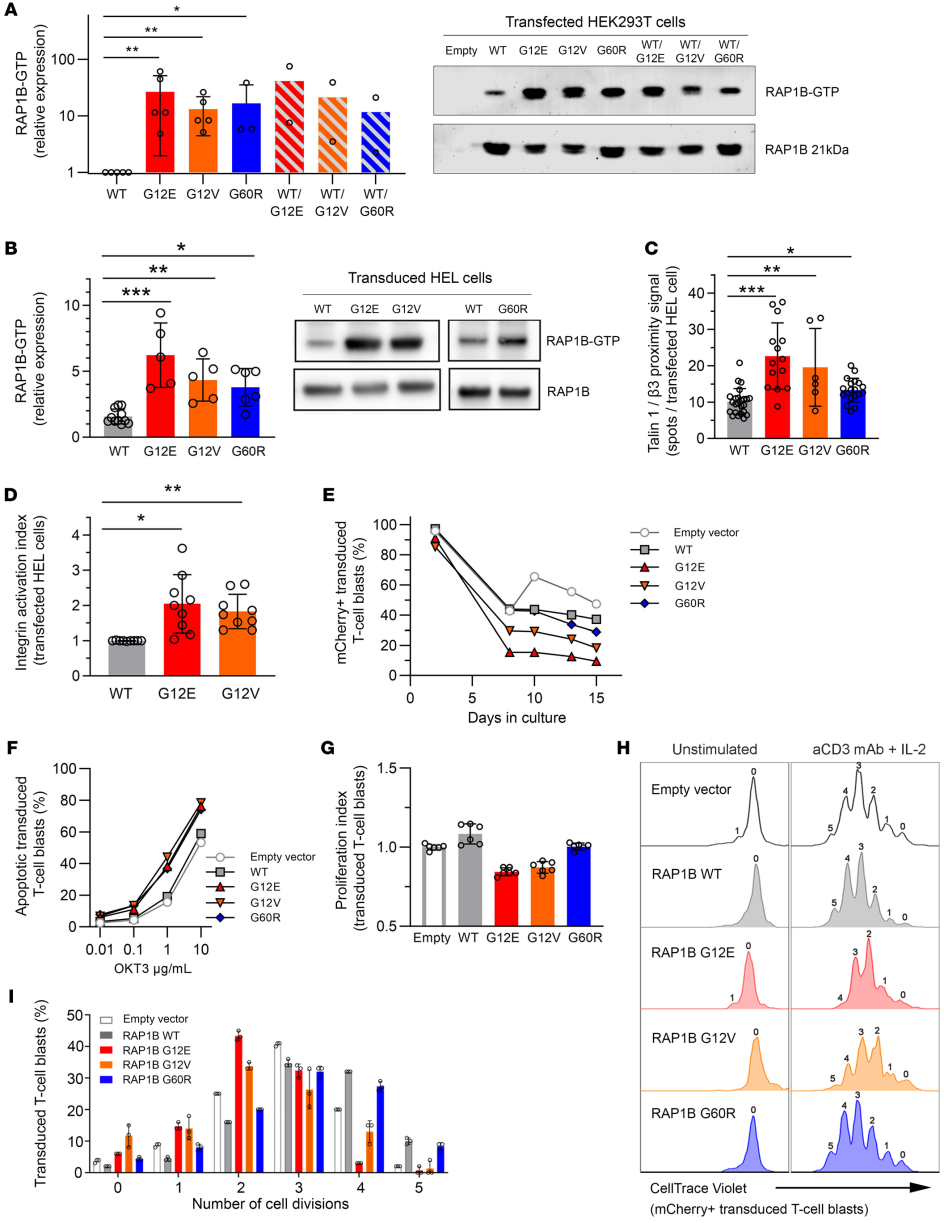
RAP1B-G12E, -G12V, and -G60R variants in overexpression models. (**A**) RAP1B-GTP activation evaluated by Western blotting after pull-down assay. HEK293T cells were transfected with vectors containing *RAP1B*-WT, *RAP1B* variants, or equimolar *RAP1B*-WT and *RAP1B* variant combinations. Values were compared with *RAP1B*-WT transfected cells (set to 1). Data from 5 independent experiments. (**B**) RAP1B-GTP activation in *RAP1B*-transduced HEL cells compared with nontransduced HEL cells (set to 1). Data from 5 independent experiments. (**C**) Talin-1/β_3_ integrin association was determined by Duolink proximity ligation assay in transfected HEL cells. Graph represents the relative number of fluorescent dots per cell in comparison with nontransfected HEL cells (set to 1). At least 50 transfected HEL cells per condition; data from 8 independent experiments. (**D**) Integrin activation in *RAP1B*-transfected HEL cells evaluated by flow cytometry measuring Oregon Green 488–labeled fibrinogen binding in unstimulated conditions. Graph represents the normalized integrin activation index, calculated as the ratio between MFI of each studied variant compared with *RAP1B*-WT transfected HEL cells. Data from 9 independent experiments. (**E**) Analysis of *RAP1B*-transduced T cell blasts over time. Cells were cultured in medium containing 15% DC-FBS and IL-2 at 100 U/ml. (**F**) Activation-induced cell death or apoptosis in *RAP1B*-transduced T cell blasts in response to increased concentrations of anti-CD3 antibody (OKT3) after 6 hours of stimulation. (**G**) Proliferation of *RAP1B*-transduced T cell blasts in the presence of 2.5 μg/ml coated anti-CD3 antibody and 100 U/ml IL-2, performed in triplicate. Graph represents the normalized proliferation index of transduced cells at days 3 and 4 compared with nontransduced and *RAP1B*-WT transduced cells. (**H**) Representative overlaid FACS histograms showing cell divisions by CellTrace Violet staining of transduced T cell blast with lentivirus expressing empty vector (light gray), RAP1B-WT (gray), RAP1B-G12E (red), RAP1B-G12V (orange), and RAP1B-G60R (blue) variants at day 4 after stimulation. (**I**) Graph corresponds to the distribution of transduced T cell blasts in different cell divisions calculated from FACS histograms in **H**. Error bars represent SEM. Statistical significance was determined for **A** by Mann-Whitney *U* test and for **B**–**D** by 1-way ANOVA, followed by Dunnett’s multiple-comparisons test. *P < 0.05; **P < 0.01; ***P < 0.001.

We next sought to study the impact of the G12E variant in human cell lines. We included also RAP1B-G12V and -G60R, which had been identified in patients with ST in the course of our investigations (5, 24). Of note, the G12V variant had been reported previously to lead to a constitutively activated protein (24). First, we determined RAP1B activation by measuring relative RAP1B-GTP abundance through pull-down assay. RAP1B activation was increased in HEK293T cells transfected with vectors containing G12E, G12V, or G60R or cotransfected with equimolar combinations of *RAP1B*-WT and 1 *RAP1B* variant when compared with cells transfected with *RAP1B*-WT (Figure 6A). These results suggest that the RAP1B-G12E, -G12V, and -G60R variants exert a dominant GOF effect.

We next evaluated the impact of G12E, G12V, and G60R in the human erythroleukemia (HEL) cell line, which recapitulates megakaryocytic hallmarks (25, 26), including integrin α_IIb_β_3_ activation. HEL cells expressing G12E exhibited significantly increased RAP1B activation (by 4.4-fold, *P* < 0.001) compared with cells expressing RAP1B-WT (Figure 6B), higher than cells expressing G12V (2.8-fold of RAP1B-WT activation; *P* < 0.01) or G60R (2.4-fold, *P* < 0.05). We next investigated talin-1 recruitment to integrin β_3_ in unstimulated HEL cells expressing G12E, G12V, or G60R by proximity ligation assay (PLA), which generates a fluorescent dot only when the 2 proteins are at a distance of 40 nm or less. The quantification of fluorescent dots per cell showed a significant increase in talin-1/β_3_ association in HEL cells expressing G12E (2.2-fold; *P* < 0.001), G12V (1.9-fold; *P* < 0.01), and G60R (1.3-fold; *P* < 0.05) compared with RAP1B-WT, confirming that RAP1B-G12E, -G12V, and -G60R were able to act on integrin activation without any stimulation (Figure 6C). Finally, to elucidate the consequences concerning integrin activation, we studied integrin α_IIb_β_3_ conformation in HEL cells by flow cytometry in resting conditions. Integrin α_IIb_β_3_ in HEL cells expressing G12E and G12V was abnormally activated (*P* < 0.05 and *P* < 0.01, respectively) compared with WT (Figure 6D). Taken together, these results indicate that RAP1B GOF variants G12E and G12V induce integrin α_IIb_β_3_ activation due to expression of constitutively active RAP1B.

We next assessed the functional consequences of G12E in T cell function. We investigated proximal T cell activation as well as late responses. In P1 T cells, global tyrosine phosphorylation and MAPK pathway activation (Tyr, PLCγ, ZAP-70, ERK1/2) after TCR stimulation were comparable to those of healthy controls. Furthermore, late responses such as proliferation and cytokine secretion were not altered after TCR stimulation (data not shown). Given the limited sample availability and the difficulty of maintaining the G12E somatic variant in cultured lymphocytes, we then transduced T cell blasts from HDs with lentiviral vectors expressing mCherry reporter gene and RAP1B-WT or -G12E, -G12V, and -G60R. The percentages of mCherry-expressing cells decreased over time in T cell blasts transduced with the different RAP1B GOF variants compared with WT-RAP1B or empty mCherry vector (Figure 6E), consistent with the results obtained in transduced HEL cells (Supplemental Figure 1D). This indicated that exogenous expression of RAP1B variants may impair survival and/or proliferation of cultured T cell blasts.

Activation-induced cell death, also referred to as restimulation-induced cell death, was increased in T cell blasts transduced with RAP1B-G12E, -G12V, or -G60R compared with RAP1B-WT and empty vector (Figure 6F). Cell proliferation in response to TCR stimulation was decreased in T cell blasts transduced with G12E and G12V (*P* < 0.01) or G60R (*P* < 0.05) compared with RAP1B-WT (Figure 6, G–I).

Collectively, these data demonstrate that P1’s monoallelic de novo RAP1B-G12E variant as well as the reported G12V and G60R are GOF mutations. Our results suggest that increased basal RAP1B activation may lead, through inside-out signaling, to a high integrin-affinity conformation that triggers, upon ligand interaction, downstream signaling cascades, modifying thereby lymphocyte migration, proliferation, and apoptosis as well as platelet activation.

## Discussion

We identified a de novo heterozygous single nucleotide substitution c.35G>A in *RAP1B*, p.G12E, in a 2-year-old boy with an undescribed hematological disorder associated with CID, neutropenia, and monocytopenia as well as severe thrombocytopenia and platelet dysfunction. The ubiquitously expressed and evolutionary conserved RAP1B was a strong candidate, as it mediates, through inside-out signaling, integrin activation (27), which is essential for platelet activation and lymphocyte migration (7, 8, 28). Rap1b-deficient mice present defective integrin activation, leading to abnormal platelet function and impaired development, adhesion, and homing in B cells (7, 8, 10, 28, 29). Deficiency of a single Rap1 protein is associated with an intermediate platelet phenotype, more severe for Rap1b than for Rap1a deficiency, whereas Rap1a/b–double-knockout in a murine megakaryocyte model leads to severe thrombocytopenia and platelet dysfunction (8). At the protein level, human RAP1B shares 95% sequence homology and similar intracellular and tissue distribution with RAP1A, which is encoded by a distinct gene (NCBI ID, NM_002884.4) at 1p13.3 (29, 30). However, in human hematopoietic cells, RAP1B expression is 2.5- to 4-fold higher than RAP1A expression (31, 32), and different posttranslational modifications determine cellular localization (33), suggesting that the 2 proteins are not functionally redundant.

RAP1B belongs to the superfamily of GTPases, molecular switches that regulate a plethora of cellular events (34). These small (20–30 kDa) monomeric proteins switch between an inactive GDP-bound OFF state and an active GTP-bound ON state and require GTPase activating proteins to enhance intrinsic GTPase activity and guanine exchange factors to efficiently stimulate GDP dissociation and hence GTP entry to switch them on (35). The interaction between residues located in the P-loop and switch I and II functional regions with the γ-phosphate groups of GTP, GDP, and Mg^2+^ induces a conformational change in the GTPase, thereby modifying its affinity for putative effector molecules that, upon their interaction, trigger downstream pathways (33, 34). G12 is located in the P-loop within the GTP-binding active site (Figure 1, C–G) (24) and is highly conserved across species and other small GTPases that orchestrate cell signaling, trafficking, and morphology pathways with exquisite temporal and spatial specificity while sharing a high sequence similarity. The important structural and functional role of G12 is further supported by an in vitro model in which Rap1-G12V gives rise to a constitutively activated protein (7, 24). Any substitution of the highly conserved small amino acids within the GTP-binding active site by amino acids with longer side chains is predicted to have a substantial impact on GTPase activity. In particular, P1’s G12E variant is expected to disturb the GTP-binding site. This may have important consequences for the position of the K16 lateral chain, which bonds with the nucleotide β and γ phosphates and on the interface that RAP1B may establish with effector proteins (24).

In public databases, only 2 missense variants had been reported in the P-loop and none in the switch I and II domains (Figure 1D) (11), highlighting the importance of these evolutionarily conserved RAP1B domains. During the course of our investigations, monoallelic germline *RAP1B* variants have been reported in patients who presented not only with thrombocytopenia, but also with additional clinical features ranging from facial dysmorphia/microcephaly and neurodevelopmental delay to abnormalities in brain, heart, kidney, and skeleton, hereafter referred to as ST: first G12V and G60R, then A59G (5, 6), and most recently, G12E (36) (see P2 to P6; Table 3). It has been speculated that these RAP1B variants may be causative for ST, but to date, no comprehensive functional studies have confirmed their causality and pathophysiological impact. A59G and G60R result in substitutions of evolutionarily conserved amino acids in the switch II domain, which changes conformation upon RAP1B activation, allowing for downstream effector target interaction (35).

P1’s hemato-immunological and bleeding phenotype is reminiscent of that of patients with mutations in genes involved in platelet and leukocyte integrin activation. For instance, mutations in *KINDLIN3*, which cause dysfunction in integrins necessary for platelet aggregation and leukocyte function, lead to severe bleeding and infections in leukocyte adhesion deficiency type III (37, 38). In platelets, engagement of integrin α_IIb_β_3_ with fibrinogen, its ligand, triggers outside-in signaling that leads to platelet activation and cytoskeleton reorganization (28, 39). Increased activation pattern of integrin α_IIb_β_3_ resulted in abnormal spreading of unstimulated P1 platelets on fibrinogen matrix. Platelet dysfunction was also suspected due to increased bleeding susceptibility in P1, which was consistent with a quantitative and qualitative platelet impairment. The abnormal integrin α_IIb_β_3_ activation pattern in P1 platelets prior to HSCT as well as the abnormal hematological and immunological parameters normalized after HSCT. This suggests that the underlying defect in P1 was intrinsic to the hematopoietic compartment. Increased RAP1B activation in P1 G12E cells suggested that G12E is a GOF variant, in line with reported in vitro data for G12V (24). Overexpression models using human HEK293T and HEL cell lines further confirmed that G12E, G12V, and G60R are GOF mutations. The activation of downstream signaling cascades (35) is enabled by active RAP1B through the recruitment of talin-1 and its subsequent binding to the cytoplasmic tail of β integrin, which results in integrin activation in hematopoietic cells (27, 39–41). When recapitulating the molecular steps required for integrin α_IIb_β_3_ activation in platelets in a RAP1B overexpression model in HEL cells, RAP1B-G12E, -G12V, and -G60R showed increased talin-1 recruitment to the intracytoplasmic β_3_ integrin domain.

Interestingly, in Rap1B, A59 and G60 form a peptide bond and are part of the conserved DxxG motif at the start of switch II (42). Although the recently described RAP1B-A59G variant was not included in our assays, it can be assumed that this variant may have effects on RAP1B function similar to those that can be extrapolated from previous studies on other small GTPase proteins. The crystal structure conformations of H-Ras were subjected to multivariate clustering analysis. The results showed that H-Ras G12V in the P-loop and A59G in the switch II region facilitate faster sampling of large-scale conformational transitions (43). In syndromic patients with neurological and dysmorphic features and musculoskeletal abnormalities, Scala et al. identified 7 RAC3 variants, including A59G, in the switch II region, recognized as a mutational hot spot among the small GTPase proteins RAC1, RAC3, and CDC42. Suppression of GTP-hydrolysis activity was observed for A59G (44). Finally, a cluster analysis on conformational changes of the GDP/KRAS complex showed that A59G and D33E highly affected the structural flexibility and internal dynamics of the switch domains and changed the free energy profiles of KRAS (45). The exquisite specificity and high evolutionary conservation of small GTPases may explain why the same substitutions in conserved residues are involved in many diseases (2, 9, 34, 46, 47).

Although RAP1B is expressed ubiquitously, P1 did not present any associated syndromic features, unlike patients with syndromic thrombocytopenia, who also exhibit neurodevelopmental retardation, growth delay, and congenital birth defects, including cardiovascular, genitourinary, neurologic, and skeletal malformations as well as microcephaly and facial dysmorphia (Table 3) (5, 6). This highlights the somatic nature of the *RAP1B* variant in P1 in contrast to the germline variants in ST patients. Interestingly, in P1, the G12E somatic variant was present in cells and tissues originating from different embryonic layers such as ectoderm (oral mucosal epithelial cells and hair follicles) and mesoderm (hematopoietic cells). However, it was absent in primary fibroblasts, which also derive from mesoderm. This suggests that the postzygotic somatic mutation may have occurred during early embryogenesis prior to gastrulation, when the blastula reorganizes into the primary germ layers (48). The somatic mosaicism in P1 not only explains the discrepancy between the severe hemato-immunological phenotype and complete absence of syndromic features, but also provides an opportunity to observe the in vivo effect of the G12E variant in P1’s various tissues and cell populations. We may speculate that a strong detrimental impact has prevented the presence of RAP1B-G12E mutated cells in diverse nonhematopoietic (neural, cardiac, musculoskeletal) tissues. The recent identification of a patient with syndromic thrombocytopenia due to a germline heterozygous RAP1B-G12E variant (P2 in Table 3) (36) clearly illustrates the deleterious effect of this variant in a wide range of tissues.

The observed GOF effect of RAP1B-G12E in P1’s cells with regard to apoptosis, proliferation, and/or survival may explain the detrimental consequences in P1’s different peripheral hemato-immunological cell compartments. Interestingly, RAP1B-G12E does not seem to have the same deleterious effect in P1’s BM cells. In a Rap1b-knockout zebrafish model, Notch signaling and its mediated specification were necessary for hematopoietic stem cell development (31) and required integrin adhesion to fibronectin through Rap1b activation (49). This is reminiscent of dominant mutations in *CXCR4*, responsible for WHIM (warts, hypogammaglobulinemia, immunodeficiency, and myelokathexis) syndrome, that lead to enhanced and prolonged β integrin activation responsible for accumulation of mature and degenerating neutrophils in the BM (50). This may explain why RAP1B GOF variants may not be as deleterious in hematopoietic precursors.

The main shortcoming of our study resides in the fact that we could not perform in vitro megakaryopoiesis and platelet formation assays on patient cells due to restricted samples from our young thrombocytopenic patient undergoing HSCT. There were rare hypolobed megakaryocytes in BM smears and biopsy. We used a method adapted from Chen et al. (51) to evaluate in vitro the formation of proplatelet-like features in Dami cells, a human megakaryocytic cell line, by transfecting RAP1B-WT, -G12E, or -G12V. While our results suggested that RAP1B variants may act on proplatelet-like formation, as the percentages of Dami cells forming proplatelet-like extensions were significantly reduced in RAP1B-G12E (*P* = 0.0011) and -G12V (*P* = 0.0057) (data not shown), we cannot extrapolate to platelet formation in the patient from this nonphysiological model using a drug (myosin II inhibitor) instead of the patient’s CD34^+^ stem cells. Further studies are thus required to precisely determine the effect of RAP1B GOF variants on human megakaryopoiesis and platelet formation. Murine Rap1A and Rap1B share 95% identity, and yet the phenotype of the isoform-specific knockout is different in mice. Therefore, investigations in a knockin mouse model may not faithfully reproduce the human phenotype. The low yield of induced pluripotent stem cell–derived (iPSC-derived) platelet preparations is another limiting factor for more comprehensive functional studies.

In conclusion, we demonstrate that P1’s monoallelic RAP1B-G12E is a de novo somatic mutation causing CID and severe thrombocytopenia associated with neutropenia and monocytopenia. We show that G12E and the previously reported RAP1B-G12V and -G60R are dominant GOF variants leading to increased RAP1B activation and integrin activation in hematopoietic cells. The activated integrin pattern may trigger, through outside-in signaling, downstream pathways involved in cytoskeletal remodeling and migration in hematopoietic cells, including platelets, lymphocytes, and BM precursors. The impact of this overactivation varies among distinct cell types and seems to have differential — beneficial, neutral, or detrimental — effects in the central hematopoietic or peripheral blood compartment.

De novo postzygotic variants that cause monogenic diseases may be underestimated and missed by genetic testing if the VAF in peripheral blood is low. If these variants appear early in embryogenesis, they may result in somatic mosaicism, which adds complexity to the clinical and biological phenotype. Our findings illustrate the phenotypic continuum of monoallelic *RAP1B* GOF variants ranging from isolated primary immunodeficiency and thrombocytopenia in a patient with somatic mosaicism to complex syndromic features in patients with germline mutations.

## Methods

### Sex as a biological variable.

We report a single male patient. However, both male and female patients carrying *RAP1B* variants have been reported (Table 3). Sex was not considered as a biological variable.

### Cells and cell culture.

See Supplemental Methods.

### Immunological analysis.

For T cell blast proliferation assays, cells were cultured without IL-2 for 72 hours for synchronization. Lymphocytes were then cultured during 3 to 7 days in complete medium alone or with 100 U/ml IL-2 in the presence of 0.1, 1, or 10 μg/ml coated anti-CD3 antibody (clone OKT3, Thermo Fisher Scientific, catalog 14-0037) or anti–CD3/CD28-coated beads (Invitrogen GmbH) as previously described (52, 53). Activation-induced cell death in response to anti-CD3 antibody (OKT3) stimulation was measured as previously described (54) in transduced T cell blasts. Apoptotic cells were analyzed after 6 hours of stimulation with increased concentrations of anti-CD3 antibody. Analyses of TCR stimulation, cytokine secretion, and calcium flux were performed as previously described (52, 53, 55). For B-LCL cell proliferation analysis, cells were washed 3 times in PBS and starved overnight for synchronization in RPMI medium supplemented with 0.1% FBS. Then, B-LCL cells were labeled with CellTrace Violet dye (Life Technologies, catalog C34557), cultured for 3 and 6 days in complete medium, and harvested. Cell proliferation was assessed using CellTrace Violet dilution by flow cytometry according to the manufacturer’s instructions. Proliferation index was calculated using FlowJo2 software, version 10.7.1, as the total number of divisions/cell that went into division. B-LCL cell cycle assays were performed using double staining with an anti-BrdU monoclonal antibody and propidium iodide (PI) as previously described (56). For B-LCL cell apoptosis, cells were synchronized overnight in RPMI medium supplemented with 0.1% FBS. Afterwards, B-LCL cells were cultured for 6 hours, 12 hours, and 24 hours in complete RPMI medium. Then cells were harvested, and apoptotic and necrotic cells were assessed using annexin V^+^ (BioLegend, catalog 640906) and 7-AAD^+^ (BioLegend, catalog 420403) staining by flow cytometry according to the manufacturer’s instructions.

### Molecular genetic assays.

See Supplemental Methods.

### WES.

WES was performed in trio on gDNA extracted from whole blood from P1 and his parents. For details, see Supplemental Methods.

### Gene-targeted sequencing.

*RAP1B* c.35G>A (G12E) VAF was determined by NGS in a targeted 2-PCR approach in gDNA samples as previously described (57). For details, see Supplemental Methods.

### Immunostaining and cell sorting.

Cell staining and flow cytometry–based phenotypic analyses of PBMCs were performed according to standard flow cytometry methods. For integrin subunit and CXCR4 receptor analysis, PBMCs were stained with anti-CD11a FITC (β2 subunit of LFA-1, clone HI111, BioLegend, catalog 301206), anti-CD18 phycoerythrin (PE) (α2L subunit of LFA-1, clone 1B4/CD18, BioLegend, catalog 373408), anti-CD29 PE (β1 subunit of VLA-4, clone TS2/16, BioLegend, catalog 303004), and/or anti-CD49d APC (α4 subunit of VLA-4, clone 9F10, BioLegend, catalog 304308) as previously described (58) and with anti-CXCR4 BV421 (clone 12G5, BD Biosciences, catalog 566282) fluorescent-conjugated antibodies. For cell-sorting details, see Supplemental Methods.

### B-LCL cell spreading.

For B-LCL cell spreading, B-EBV cells were cultivated in complete RPMI and activated with soluble IL-4 (10 ng/ml) for 24 hours. Coverslips were coated in PBS with anti-CD44 antibody for 3 hours (catalog 103046, BioLegend). Cells were placed in warm media and incubated on coverslips for 24 hours at 37°C. After incubation, cells were washed with PHEM buffer 1× and fixed in formaldehyde 4%/glutaraldehyde 0.2%/Triton X-100 0.5% in PHEM buffer 2× for 12 minutes at room temperature. PHEM buffer 1× was obtained after dilution of PHEM 2× (120 mM PIPES, 50 mM HEPES, 20 mM EGTA, 4 mM Mg acetate dissolve in Milli-Q H_2_O adjusted to pH 7.3 with NaOH) with Milli-Q H_2_O.

Cells were stained in PBS with 1% FBS for 30 minutes at room temperature using phalloidin AF647 (1:300, catalog A30107, Thermo Fisher) or anti–α-tubulin (1:500) with coupled secondary antibody (1:1,000, AF488, catalog 16-237, Sigma-Aldrich) and DAPI (1:1,000, catalog D1306, Thermo Fisher) for actin cytoskeleton. Mowiol mounting media (pH 7.4) was made with 6 g glycerol (analytical grade), 2.4 g Mowiol 4-88 (Calbiochem, catalog 475904), 6 ml Aqua dest, 12 ml 0.2 M TRIS buffer pH 74, and 2.5% DABCO (anti-bleaching reagent 1,4-diazabicyclo[2.2.2]octan; Fluka, catalog 33480). The solution was stirred for 4 hours and incubated for 2 hours at room temperature, then 10 minutes at 50°C, centrifuged at 5,000 *g* for 15 minutes, and supernatant collected. Slides were mounted with Mowiol and analyzed by confocal microscopy (Leica SP8).

Image analysis was performed using Fiji, version 2.7.0, sing maximum intensity Z projection of confocal stacks with an applied γ of 0.6 for better cell shape visualization.

### Migration assays.

PBMC migration assay was performed using Incucyte ClearView 96-well Chemotaxis Plate (Essen BioScience, catalog 4582), 100 mg/ml fibronectin coating (Sigma-Aldrich, catalog P-4707), and 1,500 ng/mL SDF-1α (CXCL12) (R&D Bio-Techne, catalog 350-NS-050) according to the Chemotaxis Cell Migration General Protocol (Essen BioScience). Scheduled images were sequentially acquired every hour using ×10 magnification. Optimized definition parameters in IncuCyte S3 Live-Cell Analysis Instrument were used to generate masks, process images, and create graphs. Migration rate was evaluated with the IncuCyte Live Cell Analysis System (IncuCyte S3, Essen Bioscience) using the normalized area (0 to 1) corresponding to the cells in the upper reservoir over time. Primary fibroblast migration assay was performed using ImageLock 96-well microplate (Essen BioScience, catalog 4379) and IncuCyte S3 Live-Cell Analysis Instrument according to IncuCyte ZOOM 96-Well Scratch Wound protocol and the manufacturer’s instructions. Age-matched controls and P1’s primary fibroblasts were seeded in 96-well culture plates and cultured until confluency. A scratch was made, plates were then installed in the IncuCyte ZOOM system, and scheduled images at ×10 magnification were taken in each well every hour. Optimized scratch wound definition parameters in the IncuCyte S3 Live-Cell Analysis Instrument were used to generate masks, process images, and generate data. Scratch closure rate was evaluated with IncuCyte software, expressed as percentages of relative wound density and wound confluency.

### Expression vectors.

For cloning details, see Supplemental Methods.

### Preparation of washed platelets.

Venous blood from HDs or patient was collected in 10% ACD/A buffer (75 mM sodium citrate, 44 mM citric acid, 136 mM dextrose, pH 4.5). Platelets were washed as previously described (59) in the presence of apyrase (100 mU/Ml; Sigma-Aldrich, catalog A6535) and prostaglandin E1 (1 μM; Sigma-Aldrich, catalog P5515) to minimize platelet activation. Platelet counts were adjusted to 3 × 10^8^ platelets/mL in Tyrode’s buffer (137 mM NaCl, 2 mM KCl, 0.3 mM NaH_2_PO_4_, 1 mM MgCl_2_, 5.5 mM glucose, 5 mM *N*-2-hydroxyethylpiperazine-N0-2-ethanesulfonic acid, 12 mM NaHCO_3_ and 2 mM CaCl_2_, pH 7.3).

### Platelet spreading analysis.

Glass coverslips were coated with 100 μg/mL human fibrinogen in PBS at 4°C overnight and then blocked with 5% BSA. Washed platelets (3 × 10^6^ platelets) were plated to fibrinogen-coated glass coverslips in the presence of apyrase (5 U/mL) and indomethacin (4.5 μM) in order to prevent platelet amplification and incubated for 30 minutes at 37°C. After washing, platelets were fixed for 15 minutes with 4% paraformaldehyde, permeabilized with 0.2% Triton X-100, and blocked with 1% BSA; platelet morphology was detected by labeling cytoskeletal F-actin using Alexa Fluor 488–phalloidin (0.3 μM). Finally, the coverslips were mounted to slides and immunofluorescence images were acquired on an epifluorescence microscope (Nikon, Eclipse 600). Recorded images were analyzed using ImageJ software (NIH).

### Platelet TEM.

Washed platelets were fixed by incubation for 1 hour at room temperature with 1.25% glutaraldehyde in 0.1 M phosphate buffer, pH 7.2, centrifuged for 10 minutes at 1,100 *g*, and washed once in phosphate buffer. Platelets were kept in 0.2% glutaraldehyde at 4°C until processing for standard TEM analysis of platelet morphology, as previously described (60).

### Talin-1 to β_3_ integrin association by PLA.

10^5^ HEL cells expressing RAP1B-WT, -G12E, or -G12V were plated on fibrinogen-coated coverslips (10 μg/mL) for 30 minutes at 37°C. Adherent cells were fixed with 4% paraformaldehyde in cytoskeleton buffer (0.1 M PIPES, 2 M glycerol, 1 mM EGTA, 1 mM MgCl_2_ pH 6.9) for 15 minutes, then permeabilized for 5 minutes in the same buffer containing 0.2% Triton X-100. Fixed cells were incubated overnight at 4°C with the monoclonal antibody directed against integrin α_IIb_β_3_ (clone P2; 2 μg/mL) and the rabbit polyclonal antibody directed against talin-1 (5 μg/mL). PLAs were performed according to the manufacturer’s instructions (Duolink InSitu, Sigma-Aldrich) using oligonucleotide-coupled secondary antibodies against mouse and rabbit primary antibodies (PLA probes). When a pair of PLA probes has bound to primary antibodies in close proximity (<40 nm), a fluorescent dot is observed by epifluorescence microscope (Nikon, Eclipse 600). The number of fluorescent dots per cell was quantified using ImageJ software in at least 50 transfected mCherry-positive HEL cells.

### Integrin α_IIb_β_3_ activation in platelets and HEL cells.

Activation of integrin α_IIb_β_3_ in platelets was evaluated in diluted whole blood (1/10 in Tyrode’s buffer) after incubation with FITC anti-human activated α_IIb_β_3_ integrin (PAC1) and PE–anti-human CD42b (clone HIP1) for 20 minutes at room temperature according to the manufacturer’s instructions. The samples were analyzed directly by flow cytometry (Accuri C6 Plus flow cytometer, BD Bioscience). Platelets were identified from other blood cells by their specific marker, CD42b. The activation of integrin α_IIb_β_3_ in RAP1B-expressing HEL cells was evaluated by quantification of soluble fibrinogen binding to cells. Briefly, 5 × 10^5^HEL cells were incubated for 10 minutes at room temperature with 20 μg/mL Oregon Green^488^–labeled human fibrinogen (Invitrogen, catalog F-7496). After arrest of the labeling by dilution, fibrinogen binding was determined by flow cytometry (Accuri C6 Plus flow cytometer) in mCherry-positive cells.

### RAP1B expression.

Washed platelets (3 × 10^8^/mL) were lysed in Laemmli sample buffer (10 mM HEPES, 2% SDS, 10% glycerol, and 5 mM EDTA). Proteins were reduced by incubation with 25 mM DTT, then separated by electrophoresis using NuPage 4%–12% Bis-Tris Protein gel (Invitrogen) and transferred to nitrocellulose membrane, which was incubated with the primary antibodies rabbit anti-human RAP1B (0.3 μg/mL) or mouse anti-CD41 (0.2 μg/mL, clone SZ22), used as loading controls for normalization. Immunoreactive bands were visualized with enhanced chemiluminescence detection reagents (ECL) using a G:BOX Chemi XT16 Image System and then quantified using Gene Tools, version 4.03.05.0 (Syngene).

### RAP1B activation.

RAP1B activation was analyzed in P1 and HD B-LCL, transfected HEK293T cells, and transduced HEL cells. HEK293T cells were transfected with pLVX-EF1α-IRES-mCherry RAP1B-WT, -G12E, -G12V, and -G60R vectors using the jetPRIME Transfection Kit (Polyplus, catalog 114-15). Cotransfections were performed using equimolar 1:1 combinations of RAP1B-WT with each RAP1B-G12E, -G12V, and -G60R vector. Forty-eight hours after transfection, more than 85% of HEK293T transfected cells were mCherry positive. HEL cells were transduced with viral supernatant containing vectors for RAP1B-WT, -G12E, or -G12V in the presence of 6 μg/mL polybrene. Two weeks after transduction and expansion, transduced HEL cells were sorted at the Flow Cytometry Core Facility of Institut Necker Enfants Malades (INEM). Transduction efficiency was evaluated by scoring mCherry transgene expression by flow cytometry (Accuri C6 flow cytometer). More than 90% of transduced cells were mCherry positive and remained stable over time. RAP1B activation (RAP1B-GTP/RAP1B) was determined in B-LCL, transfected HEK293T cells, and transduced HEL cells using active Rap1 Pull-Down and Detection Kit (Thermo Fisher Scientific, catalog 1612). RAP1B was detected by Western blotting using rabbit polyclonal IgG anti-RAP1B antibody (0.3 μg/mL, Proteintech, catalog 10840-1-AP).

### Statistics.

Results are reported as mean ± SEM. Graphs and statistical analyses were performed using GraphPad Prism software, version 9.4.1 (GraphPad Software). Data were compared using 2-tailed Student’s *t* test,Mann-Whitney *U* test, or 1-way ANOVA followed by post hoc test for multiple comparisons, as indicated in the figure legends. Differences were considered significant when *P* < 0.05.

### Study approval.

The institutional INSERM and Necker-Enfants Malades Hospital Review Boards approved this study. AP-HP obtained a favorable opinion from the Comité de Protection des Personnes for this research (CPP Ile de France II) on March 11, 2015. Written, informed consent was obtained from the patient’s parents in accordance with the Helsinki Declaration. HD cells were obtained under agreement EFS C-CPSL-UNT N°20/EFS/005.

### Data availability.

Values for all data points in graphs are reported in the Supporting Data Values file.

## Author contributions

DM identified the variant and conceived the project. MBN, FA, and DM designed the experiments. MBN, FA, and EM performed most of the experiments and analyzed the data. MBN performed HEK293T, B-LCL, PBMC, and T cell blast experiments, established primary fibroblast culture, performed cell sorting and NGS analysis, generated expression vectors, and produced lentiviral supernatants. C Boussard performed cell morphology in B-LCL. FA, helped by AK, CR, and MF, performed the experiments carried out in platelets and HEL cell lines. EM performed PBMC immunophenotype and T cell blast functional experiments. CLP helped to design BM cell-sorting experiments and performed functional experiments from CD34^+^ cells. C Brouzes analyzed P1 BM smears. CP and NL performed immunophenotyping and microsatellite analysis. PP performed P1 B-LCL cell migration in microchannels. MZ performed WES. JCB performed TEM analysis of P1 platelets. DM, DB, JRG, MC, GM, and C Boussard provided medical care and treated P1. IC performed formal analysis and data visualization and contributed to writing. DM and MBN wrote the manuscript, with contributions from FA, PR, and JPDV. DM wrote the revised version of the manuscript, with contributions from MBN, FA, PR, and JPDV. All authors read and approved the manuscript. MBN and FA contributed equally to data production and are credited as co–first authors. MBN was chosen as the first name because she wrote the largest fraction of the manuscript.

## Supplementary Material

Supplemental data

Unedited blot and gel images

Supporting data values

## Figures and Tables

**Table 2:**
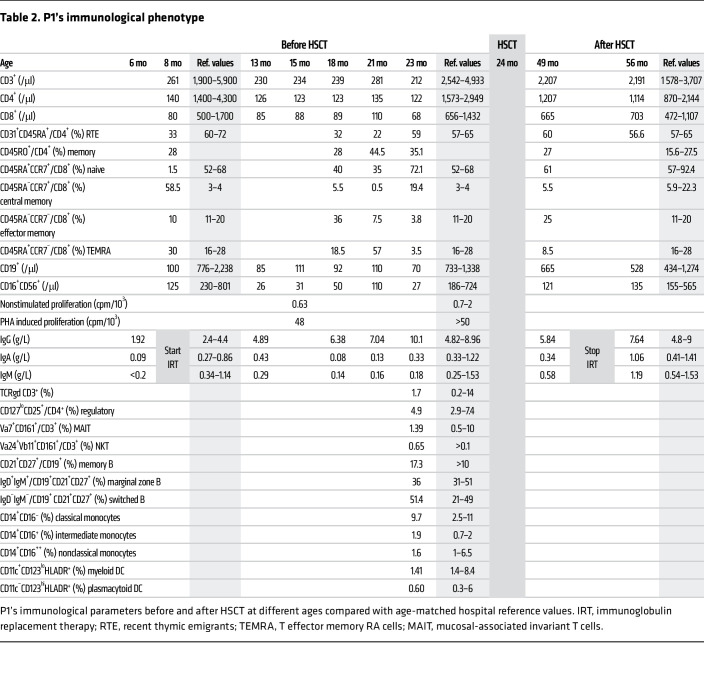
P1’s immunological phenotype

**Table 1:**
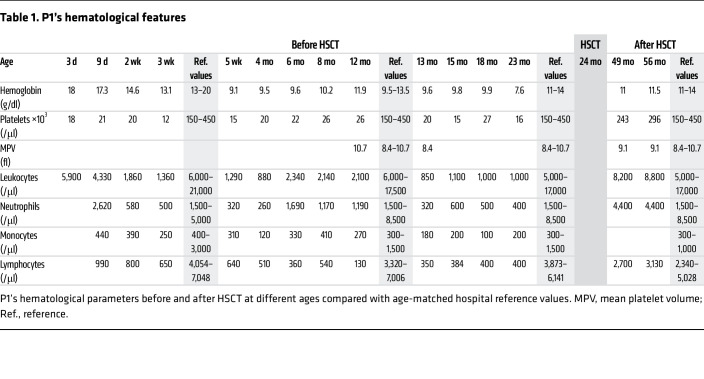
P1’s hematological features

**Table 3:**
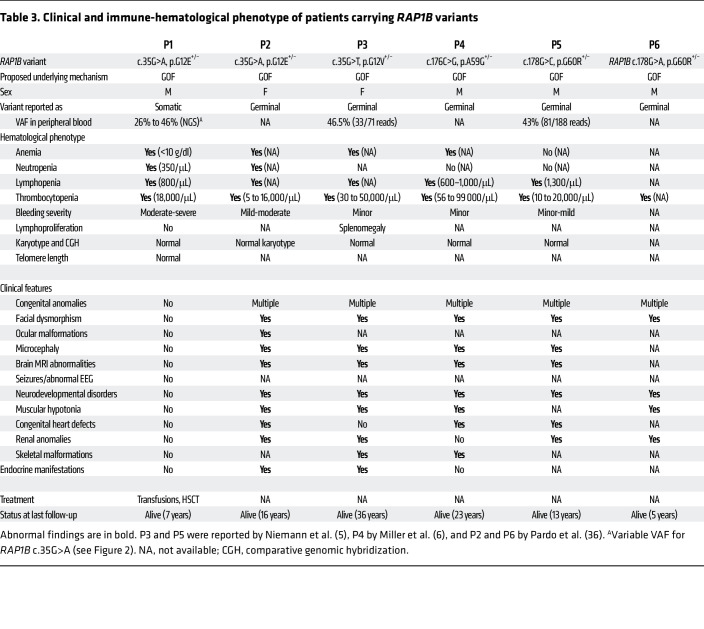
Clinical and immune-hematological phenotype of patients carrying *RAP1B* variants

## Version 1 09/03/2024

Electronic publication
