# Supplementary material for: Somatic RAP1B gain-of-function variant underlies isolated thrombocytopenia and immunodeficiency
Source: J Clin Invest. 2024 Jul 11;134(17):e169994. doi: 10.1172/JCI169994 (PMC11364392; doi:10.1172/JCI169994)
Supplement: Supplemental data [file jci-134-169994-s101.pdf]

SUPPLEMENTAL DATA

Somatic *RAP1B* gain-of-function variant underlies isolated thrombocytopenia and immunodeficiency

by Marta Benavides-Nieto et al.

Supplemental Figure 1

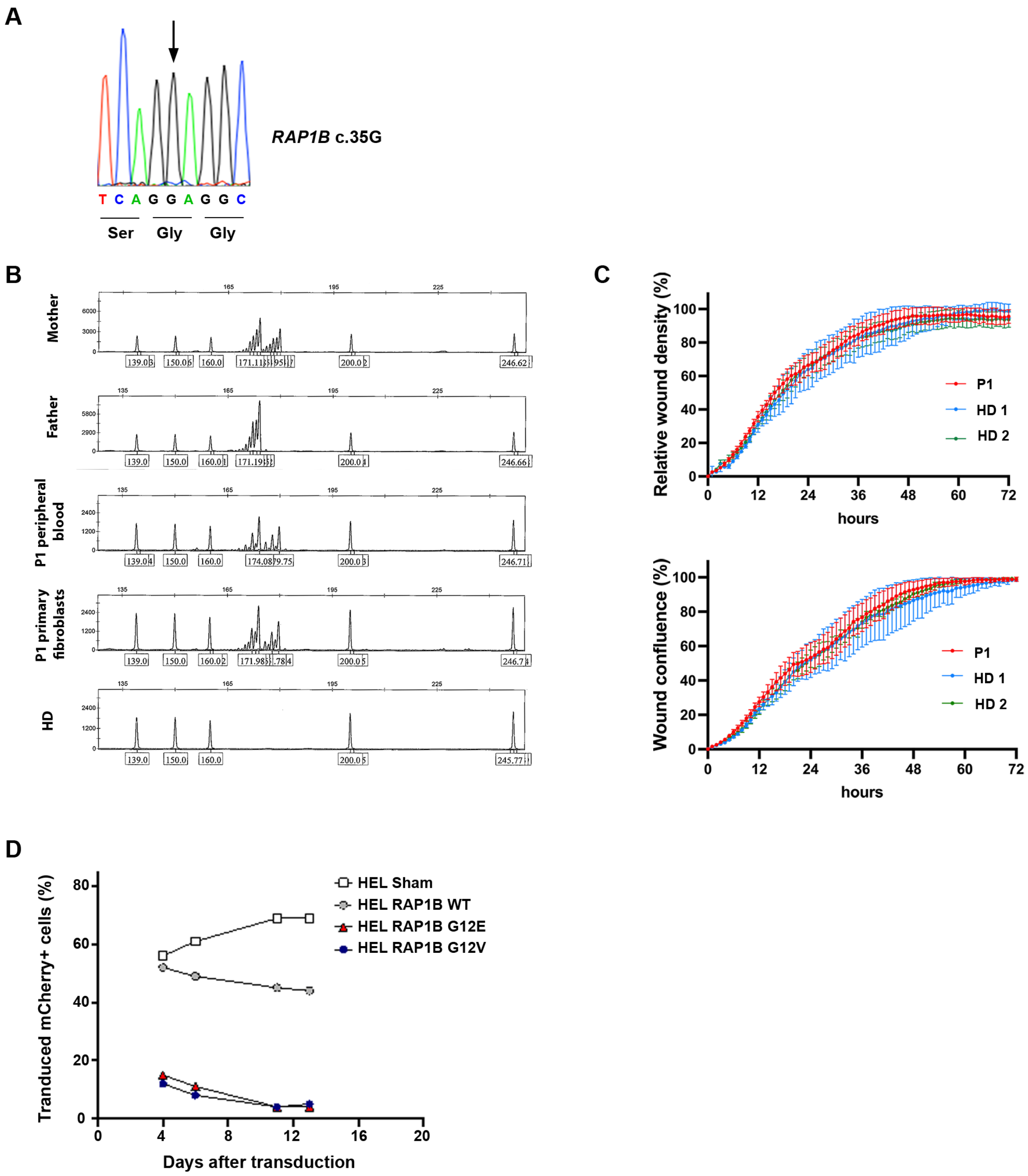

Supplemental Figure 1: (**A**) Sanger sequencing shows that P1 primary fibroblasts do not carry the single nucleotide substitution c.35G>A in *RAP1B*. (**B**) Microsatellite analysis at Munc13-4 locus from gDNA obtained from P1 whole blood and fibroblasts, P1's parents and healthy control (HD) samples confirms the identity of P1 primary fibroblasts. (**C**) Migration of P1 primary fibroblasts and age-matched controls (HDs) analyzed by using Scratch assay using the IncuCyte ZOOM 96-Well Scratch Wound. Performed in triplicates. (**D**) Percentage of transduced (mCherry+ expressing) HEL cells over time using pLVX-IRES-mCherry carrying RAP1B open reading frame (WT sequence or harboring G12E, G12V or G60R variants).

Supplemental Table 1: Etiological investigations prior to HSCT

- Negative maternal serologies for Toxoplasmosis, HIV, HAV and HBV, positive for Rubella.
- PCR in blood: Parvovirus, cytomegalovirus (CMV), Epstein-Barr virus (EBV), VIH: negative.
- Normal transfontanellar and abdominal ultrasound.
- Bone marrow (BM, 1 week): low richness with rare megakaryocytes.
- BM smear (3 weeks): low richness with rare small and hypolobed megakaryocytes.
- BM smear (3.5 months): low richness, absent megakaryocytes, diminished myeloid and granuloid lineages.
- BM smear (7 months): low richness, absent megakaryocytes.
- BM smear (18 months): low richness, presence of elements at all stages of maturation, predominance of the granular lineage, absence of atypical cells. Rare hypolobed megakaryocytes.
- BM biopsy (3.5 months): expansion of hematogones, relative megakaryocytic hyperplasia with maturation disorders. Granular and erythroblastic hypoplasia without major maturation disorder.
- BM Karyotype (1 week and 3.5 months): 46, XY, normal.
- Peripheral blood karyotype (7 months): 46, XY, normal.
- BM search for monosomy 5/del5q, 7/del7q (3.5 months): negative.
- Neutrophil functional tests (oxidative explosion, production of oxygen reactive species, and spontaneous and fMLF & sera-induced movement (22 and 23 months)): normal.
- Alloimmunization tests (1 month) including paternal neutrophil groups incompatibility analysis, anti-granulocytic antibodies and platelet Coombs: negative.
- Anti-transglutaminase and anti-enterocytes IgG (6 months): negative
- T cell repertoire, percentage of Vα7.2 (8 months): normal.
- PROMIDIS assay(1) (14 months): normal.
- ADA (adenosine deaminase) and PNP (purine nucleoside phosphorylase) levels (8 months): normal.
- Wiskott–Aldrich Syndrome Protein *WASP* gene (11 months): WT sequence.
- *RAG* (recombination-activating genes) 1 and 2 (8 months): WT sequence.
- Telomere restriction fragment (TRF) measurement: normal.
- Comparative genomic hybridization (CGH) array: normal.
- MPL sequence in BM (11 months): WT sequence.
- Thrombopoietin levels (1 month): normal.
- PNH (paroxysmal nocturnal hemoglobinuria) clone in neutrophils, monocytes and red blood cells (5 months): absent.
- X-ray left hand (2 months): normal.
- Genetic test for Fanconi Anemia in BM, peripheral blood and primary fibroblasts: negative.
- Chest X ray (6 months): thymus shade present, no osseous anomalies.

In brackets: age at analysis.

Supplemental Table 2: Variant allele frequency (VAF) and percentage of RAP1B $^{WT/G12E}$ mutated cells in different P1 samples analyzed by NGS and/or EditR(2)

	**Nature of the sample**	**Reads**	***RAP1B* c.35G>A VAF**		***RAP1B$^{WT/G12E}$* cells**
			**By *NGS***	***By EditR***	
P1's mother	Peripheral blood	**78 265**	0.05%		0.1%
P1's father	Peripheral blood	**127 862**	0.06%		0.12%
P1	Peripheral blood (M8)	**619 829**	**39.78%**	**39%**	**79.6%**
P1	Peripheral blood (M12); used for WES	**257**	**42%**		**84%**
P1	Peripheral blood (M18)	**172 163**	**37.14%**		**74.3%**
P1	Peripheral blood (M21)	**151 520**	**26.25%**		**52.50%**
P1	Peripheral blood (M24)	**88 889**	**21.94%**		**43.9%**
P1	Primary fibroblasts	**149 988**	0.08%		0.16%
P1	Hair follicles	**111 605**	**46.77%**	44%	**93.5%**
P1	Buccal swab cells (M7 post HSCT)	**552 805**	**21.48%**	22%	**43%**
P1	B-LCL cells	**137 570**	**21.70%**		**43.40%**
P1	B-LCL cells, D0 of culture	N/A		**37.44%**	74.9%
P1	B-LCL cells, D13 of culture	N/A		**27.15%**	54.3%
P1	B-LCL cells, D18 of culture	N/A		**24.74%**	49.5%
P1	B-LCL cells, D35 of culture	N/A		**14.08%**	28.2%
P1	B-LCL cells, D40 of culture	N/A		**9.76%**	19.5%
P1	Peripheral blood CD4+ T cells (M21)	**192 472**	**18.30%**	17.49%	**36.6%**
P1	Peripheral blood CD8+ T cells (M21)	**59 812**	**19.40%**	19.78%	**38.8%**
P1	Peripheral blood CD19+ B cells (M21)	N/A		**37.87%**	75.7%
P1	Peripheral blood CD56+ NK cells (M21)	**126 529**	**18.86%**	20.1%	**37.7%**
P1	Peripheral blood bulk (M21)	N/A		22%	44%
P1	BM HSPC CD34+ (1week)	N/A		**38%**	76%
P1	BM ProB cells (1week)	**91 810**	**45.55%**	39%	**91.1%**
P1	BM B cells (1week)	**50 462**	**42.67%**		**85.3%**
P1	BM Myeloid cells (1week)	**74 107**	**45.57%**	48%	**91.1%**
P1	BM bulk (1week)	**149 833**	**45.98%**		**92%**
P1	BM HSPC CD34+ (M7)	**326 286**	**42.25%**	42%	**84.5%**
P1	BM ProB cells (M7)	**166 111**	**41.01%**	44%	**82%**
P1	BM B cells (M7)	**65 981**	**49.72%**	47%	**99.4%**
P1	BM Myeloid cells (M7)	**54 860**	**44.48%**	47%	**89%**
P1	BM bulk (M7)	**366 827**	**44.83%**	43%	**89.7%**
P1	BM CD34+ (M7) proliferation D15	**46 661**	**41.24%**		**82.5%**
P1	B-LCL cells cycle G1	N/A		**34%**	68%
P1	B-LCL cells cycle S	N/A		**49.5%**	99%
P1	B-LCL cells cycle G2	N/A		**29%**	58%
P1	B-LCL cells bulk	N/A		**34%**	68%
P1	B-LCL cells, proliferation & apoptosis	N/A		**35%**	70%
P1	B-LCL cells, proliferation & apoptosis	N/A		**35%**	60%
P1	B-LCL cells, proliferation & apoptosis	N/A		**1%**	2%
	B-LCL cells, proliferation & apoptosis	N/A		**0%**	0%
	B-LCL cells, immunofluorescence	N/A		**37%**	74%

In brackets: age or time after HSCT at sampling. WES: whole exome sequencing

Supplemental Table 3: Primers

**Primer**	**Sequence of the primer**	**Size of the PCR product (bp)**
RAP1B_gDNAF1	TCTATCCTGGATCGGGCTCA	496
RAP1B_gDNAR1	TGGAGTCAATGTTCCAGAGAGAA	
RAP1B_cDNAF EcoRI	GAATTCTGCATCATGCGTGAGTATAAG	555
RAP1B_cDNAR BamH1	GGATCCTTAAAGCAGCTGACATGAT	
NGS MR3-GOI-RAP1B_F	GCAGCGTCAGATGTGTATAAGAGACAG<br>ATGCGTGAGTATAAGCTAGTCGT	405
NGS MR4-GOI-RAP1B_R	TGGGCTCGGAGATGTGTATAAGAGACAG<br>CTGGCTCTGAAATGCGAATGC	
RAP1B-G12Vmutagenesis_F	GTCGTTCTTGGCTCAG**t**AGGCGTTGGAAAGTC	
RAP1B-G12Vmutagenesis_R	GACTTTCCAACGCCT**a**CTGAGCCAAGAACGAC	
RAP1B-G60Rmutagenesis_F	GAAATCTTGGATACTGCA**c**GAACGGAGCAATTTACAG	
RAP1B-G60Rmutagenesis_R	CTGTAAATTGCTCCGTTC**g**TGCAGTATCCAAGATTTC	

SUPPLEMENTAL METHODS

Cells and cell culture

Peripheral blood mononuclear cells (PBMCs) and bone marrow mononuclear cells (MNC) collected from P1 and healthy donors were isolated by means of gradient centrifugation on Lymphocyte Separate Medium, density 1 077 g/mL (Dutscher, # 500201, Bernolsheim, France). EBV-transformed lymphoblastoid B-cell lines (B-LCL) were obtained from P1's and controls' PBMCs by the Centre de Ressources Biologiques (CRB-ADN, Institute Imagine, Paris). Cells were grown in RPMI 1640 Medium, GlutaMAX™ Supplement medium (Life Technologies, # 61870044, South San Francisco, USA) supplemented with 10% heat-inactivated FBS (fetal bovine serum) and 1% Penicillin-Streptomycin (Life Technologies, # 15070063). Primary fibroblasts were obtained through culture of skin biopsy from P1 and cells were grown in RPMI medium supplemented with 10% FBS and 1% Penicillin/Streptomycin. Control primary fibroblasts were from age-matched healthy donors.

HEK293T cells (CRL-11268) and HEL cells (HEL 92.1.7 (ATCC TIB-180)) were from American Type Culture Collection (Manassas, VA, USA).

Molecular genetic assays, whole-exome sequencing and gene targeted sequencing

Genomic DNA (gDNA) from P1 and control cells was extracted with the DNA isolation kit (Roche Diagnostics, # 11796828001, Basel, Switzerland). Total RNA from P1 and control cells was extracted using Life PureLink™ RNA Mini Kit (Life Technologies, # 12183025) according to the manufacturer's instructions. RNA concentration was assessed using a DS-11 FX+ spectrophotometer (DeNovix). RNA samples were treated with DNAse I (Life Technologies, # 18068015). Reverse transcription was performed using SuperScript™ II Reverse Transcriptase (Invitrogen, #18064). Polymerase Chain reactions on gDNA and complementary DNA (cDNA) were performed with designed primers (Supplemental Table 3) targeting the region around the reported mutation (*RAP1B* c.35A>G) using GoTaq® G2 Hot Start Polymerase (Promega, # M7408, Madison, USA). The PCR products were sequenced using BigDye™ Terminator v3.1 Cycle Sequencing Kit (Life Technologies, # 4337455) according to the manufacturer's instructions.

P1's whole exome sequencing (WES) was performed *in trio* with his parents on gDNA extracted from whole blood. Exome capture was performed with the Sure Select Human All Exon kit (Agilent Technologies, Santa Clara, USA). Agilent Sure Select Human All Exon (58 Mb, V6) libraries were prepared from 3 µg of genomic DNA sheared with an Ultrasonicator (Covaris) as recommended by the manufacturer. Barcoded exome libraries were pooled and sequenced with a HiSeq2500 system (Illumina, Évry-Courcouronnes, France), generating paired-end reads 130 + 130 bases. After demultiplexing, sequences were mapped on the human genome reference (NCBI build 37, hg19

version) with BWA(3). The mean depth of coverage obtained for the proband's exome libraries was >150X with > =96.6% and >=95.5% of the targeted exonic bases covered at least by respectively 15 and 30 independent sequencing reads (>=96.6% at 15X >=95.5% at 30X). Variant calling was carried out with the Genome Analysis Toolkit (GATK), SAMtools, and Picard tools, following documented best practices (http://www.broadinstitute.org/gatk/guide/topic?name=best-practices). Single-nucleotide variants were called with GATK Unified Genotyper, whereas indel calls were made with the GATK IndelGenotyper_v2. All variants with a read coverage %23 and a Phred-scaled quality %20 were filtered out. All variants were annotated and filtered with PolyWeb, an in-house-developed annotation software.

We performed next-generation sequencing (NGS) in order to determine the proportion of cells carrying the *RAP1B* c.35G>A (G12E) variant in the different gDNA samples. *RAP1B* c.35G>A (G12E) variant allele frequency (VAF) was determined by NGS in a targeted 2-PCR approach, as previously described(1), in different P1 and parents' gDNA samples. The primers framing P1's *RAP1B* c.35G>A variant (Supplemental Table 3) were designed using NCBI primer-BLAST tool(4) and synthesized by Eurofins Genomics (Ebersberg, Germany). An anchor sequence (underlined) was added to the former primers for subsequent NGS sequencing on the Personal Genome Machine (PGM, IonTorrent, Life Technologies), as previously described(1). The Q5® High-Fidelity DNA Polymerase (New England Biolabs, # M0493L, Ipswich, USA) was used according to the manufacturer's instructions, with a final reaction volume of 25 µL, 25 cycles and annealing temperature of 60°C. Successful PCR amplification was confirmed after electrophoresis in a 2% agarose gel with a band of the expected size. PCR bands were gel purified and reamplified using Advantage GC 2 polymerase (Takara, # 639114, Saint-Germain-en-Laye, France) with a final reaction volume of 25 µL, 13 cycles and annealing temperature of 68 °C and PGM sequencing primers targeting the anchor sequences (P5-i5-MR3_F 5'AATGATACGGCGACCACCGAGATCTACAC[i5]TCGTCGGCAGCGTCAGATGTG 3' and P7-i7-MR4_R 5'CAAGCAGAAGACGGCATACGAGAT[i7]GTCTCGTGGGCTCGGAGATGT 3'), where i5 and i7 represent the barcode (BCx) sequence. PCR products were purified at the expected band size and sequence on the PGM was performed according to the manufacturer's recommendations. NGS results obtained as Fastq files were analyzed. VAF was quantified by measuring the number of reads containing the reported *RAP1B* c.35G>A (G12E) variant among the total number of high-quality reads aligned to the reference sequence (Supplemental Table 2).

Cell sorting

For cell sorting, PBMCs, B-LCL and MNC from P1 and aged-matched healthy controls were stained and sorted on an Aria II system (BD Biosciences). PBMCs were stained using anti-CD3 Brilliant Violet 650™ (Sony, # RT2102340, San Jose, USA), anti-CD4 Brilliant Violet 421™ (Sony, # 357424), anti-CD8 Brilliant

Violet 510™ (Sony, # RT2323660), anti-CD19 PE Cy7 (BD Biosciences, # 560911), anti-CD56 PE (Miltenyi, # 130-113-307, San Diego, USA), APC anti-human CD11b APC (Miltenyi, # 130-110-554) antibodies, and 7-AAD (Miltenyi, # 130-111-568). After staining, PBMCs were directly sorted on an Aria II system (BD Biosciences) into CD4 T cells (CD3+ CD4+), CD8+ T cells (CD3+ CD8+), B lymphocytes (CD19+), NK cells (CD56+ CD11b-). MNCs were stained using anti-CD45 BV510 (Biolegend, # 2120180), anti-CD19 BV421 (Biolegend, # 211170), anti-CD11b APC (BD Biosciences, # 550019), anti-CD34 APC Cy7 (Biolegend, # 581 343514) antibodies, and 7-AAD (Miltenyi, # 130-111-568). After staining MNCs were directly sorted on an Aria II system (BD Biosciences) into HSCP cells (CD45+ CD34+ CD19-), ProB cells (CD45+ CD34+ CD19+), B cells (CD45+ CD34- CD19+) and myeloid cells (CD45+ CD11b+). P1's B-LCL cells were sorted on an Aria II system (BD Biosciences) into G1, S and G2/M phases, combining BrdU and PI-FITC expression.

Expression vectors

cDNA encoding full-length WT and G12E human RAP1B was amplified from control and P1's cDNA using primers flanking cDNA RAP1B sequence and including EcoRI and BamH1 enzymatic restriction sites (Supplemental Table 3). WT and G12E RAP1B transcripts were cloned in TOPO™ TA (Life Technologies, # 450641), and introduced into a pLVX-EF1α-IRES-mCherry expression vector (Clontech, #631987, Palo Alto, USA) using digestion with EcoRI and BamH1 restriction enzymes (New England Biolabs, # R0101 and R0136) and ligation with T4 DNA ligase enzyme (New England Biolabs, #15224017). The ligation product was dialysed and transformed into Top10 electrocompetent (Life Technologies, # C404052) or Stbl3™ chemically competent (Life Technologies, # C737303) bacteria. Vectors were isolated using PureLink™ HiPure Plasmid Filter Midiprep Kit (Life Technologies, # K210015) after colony subculture. pLVX-EF1α-IRES-mCherry RAP1B vectors encompassing G12V and G60R mutations were generated by directed mutagenesis using Q5 Site-Directed Mutagenesis Kit (New England Biolabs, # E0554S) according to the manufacturer's instructions. pLVX-EF1α-IRES-mCherry vector containing the RAP1B WT coding sequence was used as template. Forward and Reverse oligonucleotides were specifically generated for mutagenesis (Supplemental Table 3).

pLVX-IRES-mCherry vectors containing RAP1B WT-, G12E-, G12V-, and G60R- RAP1B cDNA were used for transient transfections and lentiviral supernatant production and transduction following standard protocols(5). The cDNA for wild-type or RAP1B variants (G12E, G12V, or G60R) inserted in pLVX-IRES-mCherry vector were transiently expressed in human erythroleukemia (HEL) cell line by nucleofection using the Neon transfection system (Invitrogen) and in HEK293T cells using jetPRIME transfection kit (Polyplus, # 114-15, Illkirch-Graffenstaden, France) according to the manufacturer's instructions.

SUPPLEMENTAL REFERENCES

1. Berland A, et al. PROMIDISα: A T-cell receptor α signature associated with immunodeficiencies caused by V(D)J recombination defects. *Journal of Allergy and Clinical Immunology*. 2019;143(1):325-334.e2.

2. Kluesner MG, et al. EditR: A Method to Quantify Base Editing from Sanger Sequencing. *The CRISPR Journal*. 2018;1(3):239–250.

3. Li H, Durbin R. Fast and accurate short read alignment with Burrows–Wheeler transform. *Bioinformatics*. 2009;25(14):1754–1760.

4. Ye J, et al. Primer-BLAST: A tool to design target-specific primers for polymerase chain reaction. *BMC Bioinformatics*. 2012;13(1):134.

5. Tiscornia G, Singer O, Verma IM. Production and purification of lentiviral vectors. *Nat Protoc*. 2006;1(1):241–245.

6. Adam F, et al. Thrombin-induced platelet PAR4 activation: role of glycoprotein Ib and ADP. *J Thromb Haemost*. 2003;1(4):798–804.
