# Supplementary material for: Somatic RAP1B gain-of-function variant underlies isolated thrombocytopenia and immunodeficiency
Source: J Clin Invest. 2024 Jul 11;134(17):e169994. doi: 10.1172/JCI169994 (PMC11364392; doi:10.1172/JCI169994)

Full unedited gel/blot for **Figure 3E**

CD41 and RAP1B for HD, P1 (pre-HSCT) and P1 (post-HSCT)

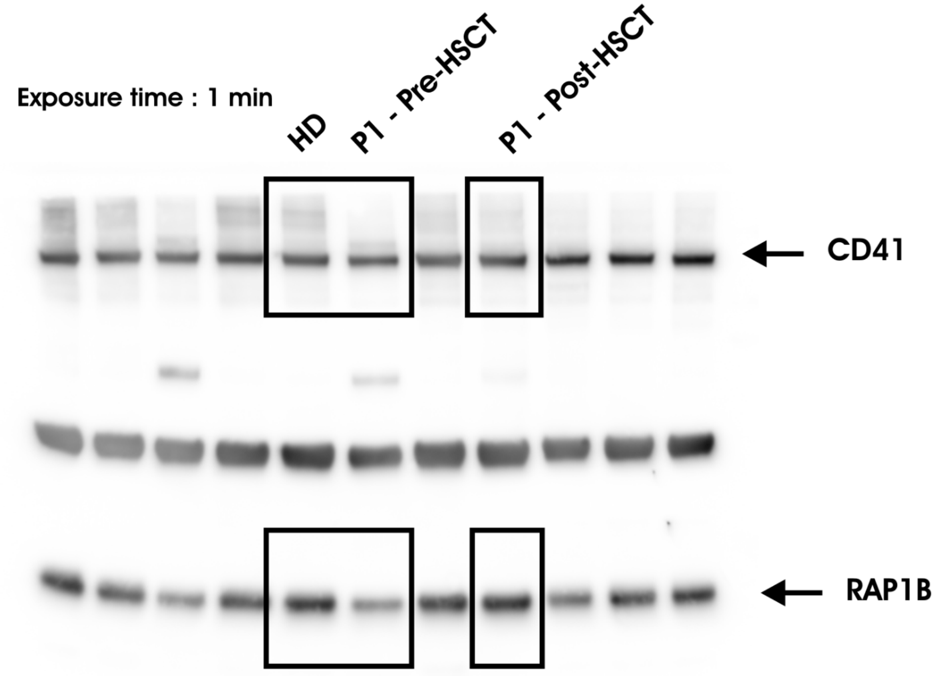

Full unedited gel/blot for **Figure 4B**

RAP1B for P1 B-LCL 80%, 14%, 4% and HD

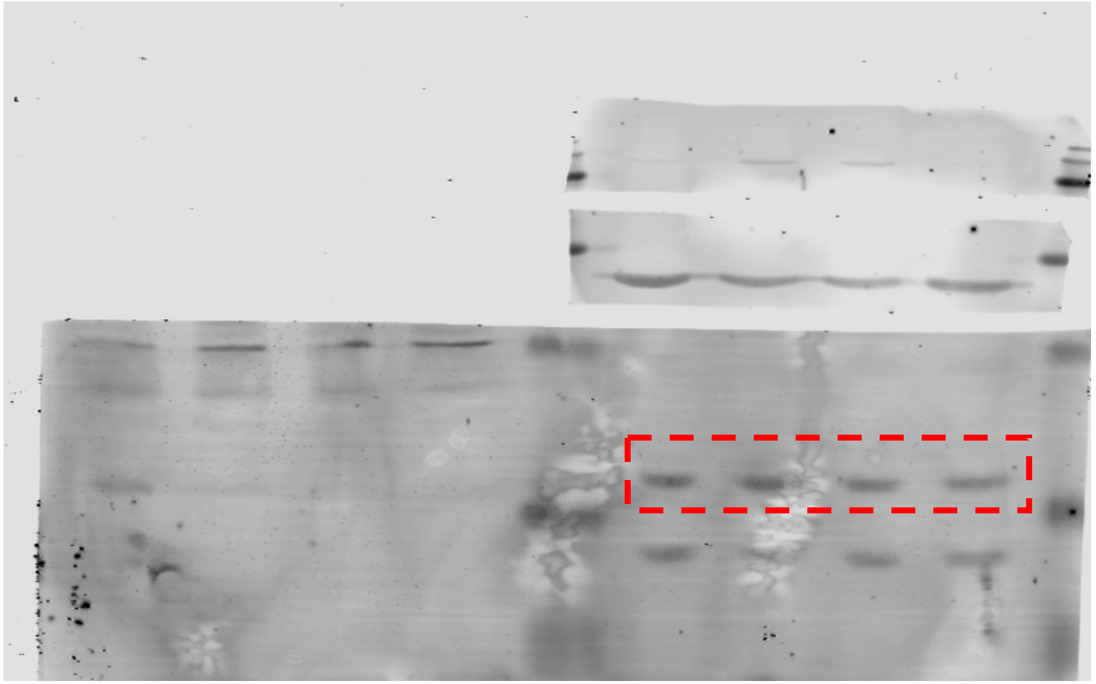

RAP1B-GTP for P1 B-LCL 80%, 14%, 4% and HD

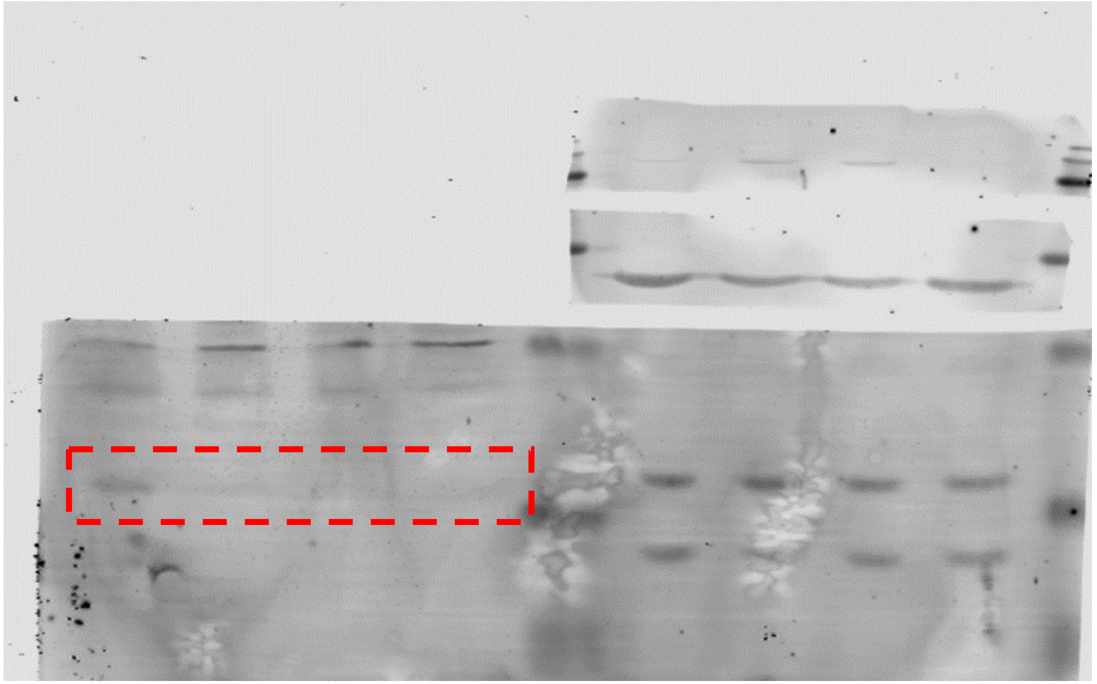

Full unedited gel/blot for **Figure 6A**

RAP1B for empty vector, RAP1B-WT, -G12E, -G12V, -G60R, -WT/G12E, -WT/G12V, -WT/G60R

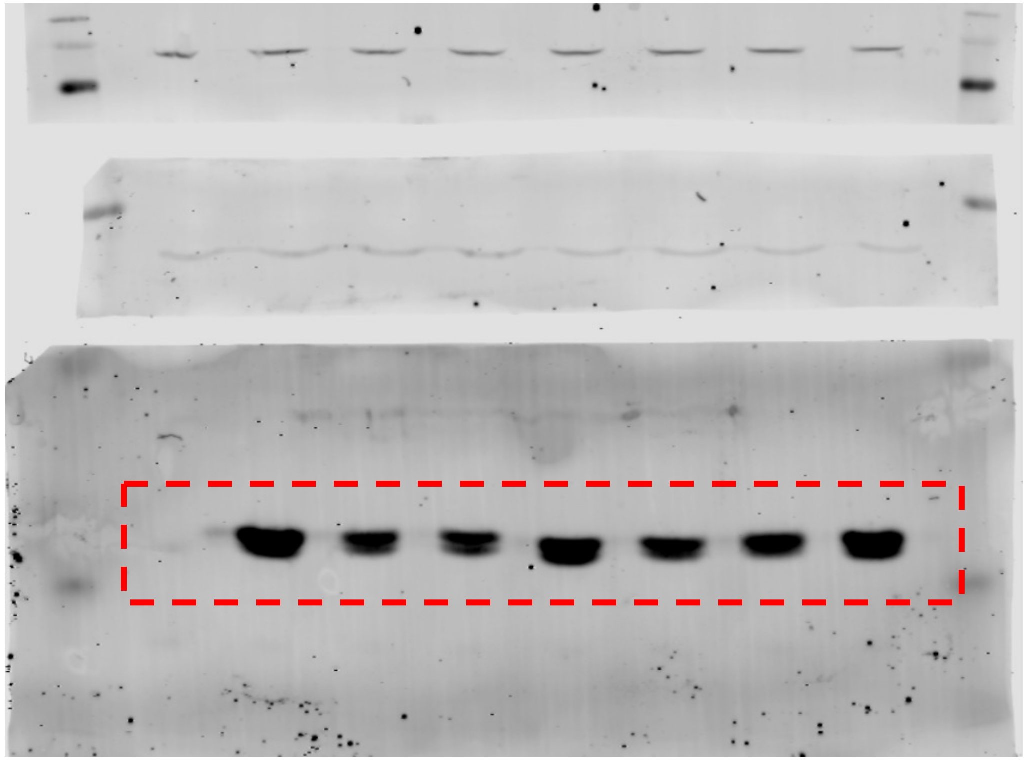

RAP1B-GTP for empty vector, RAP1B-WT, -G12E, -G12V, -G60R, -WT/G12E, -WT/G12V, -WT/G60R

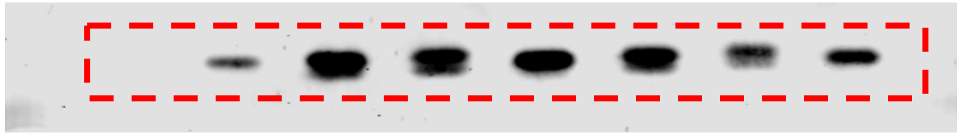

Full unedited gel/blot for **Figure 6B**

RAP1B-GTP and RAP1B for WT, G12E and G12V

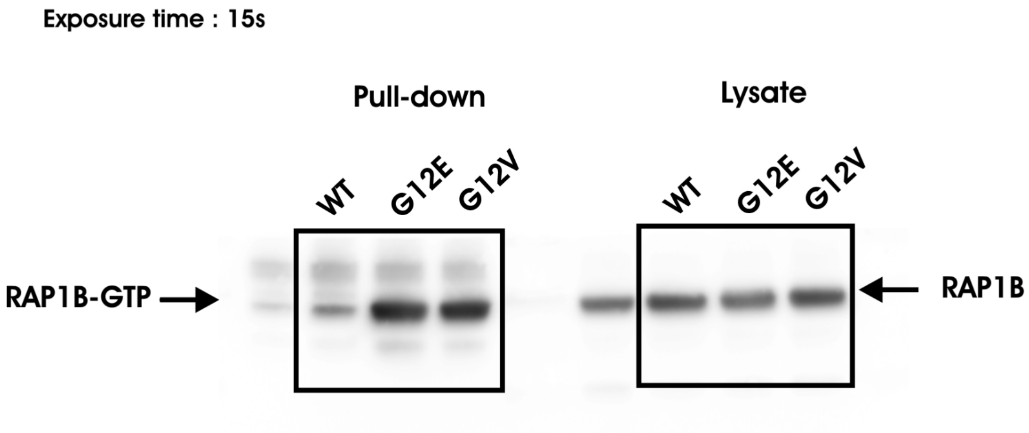

RAP1B-GTP and RAP1B WT and G60R

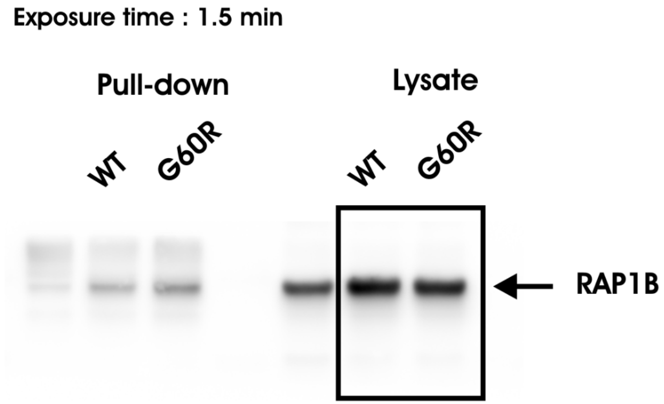

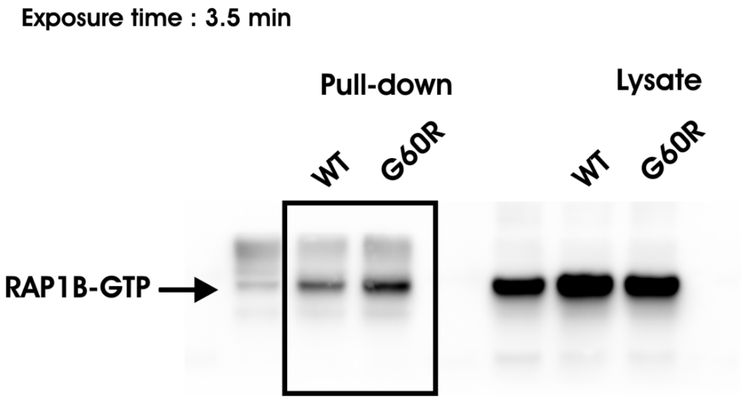

Supplement: Unedited blot and gel images [file jci-134-169994-s102.pdf]
